# The Development of Sound Localization Latency in Infants and Young Children with Normal Hearing

**DOI:** 10.1177/23312165221088398

**Published:** 2022-05-03

**Authors:** Martin Eklöf, Filip Asp, Erik Berninger

**Affiliations:** 1 Department of Clinical Science, Intervention and Technology, 27106 Karolinska Institutet, Stockholm, Sweden; 2 Department of ENT, Section of Cochlear Implants, Karolinska University Hospital, Stockholm, Sweden; 3 Department of Audiology and Neurotology, Karolinska University Hospital, Stockholm, Sweden

**Keywords:** saccade, response latency, reaction time, auditory target

## Abstract

With the advances in eye tracking, saccadic reflexes towards auditory stimuli have become an easily accessible behavioral response. The present study investigated the development of horizontal sound localization latency quantified by saccadic reflexes in infants and young children with normal hearing (0.55 to 5.6 years, *n* = 22). The subject was seated in front of an array of 12 loudspeaker/display-pairs arranged equidistantly in an arc from −55 to + 55° azimuth. An ongoing auditory-visual stimulus was presented at 63 dB SPL and shifted to another randomly selected pair at 24 occasions. At each shift, the visual part of the stimulus was blanked for 1.6 s providing auditory-only localization cues. A sigmoid model was fitted to the gaze samples following the azimuthal sound shifts. The overall sound localization latency (SLL) for a subject was defined as the mean of the latencies for all trials included by objective criteria. The SLL was assessed in 21 of 22 children with a mean of 6.1 valid trials. The SLL ranged 400 to 1400 ms (mean = 860 ms). An inverse model demonstrated a significant relationship between SLL and age (*R*^2^ = 0.79, *p* < 0.001), reflecting a distinct reduction of latency with increasing age. No partial correlation between SLL and sound localization accuracy was found when controlling for age (*p* = 0.5), suggesting that localization latency may provide diagnostic value beyond accuracy.

## Introduction

The gaze saccadic latency towards auditory targets has not been studied in infants and young children. Already at birth, humans react to auditory events by turning their heads (e.g. Morrongiello et al., 1994) or eyes (Wertheimer, 1961) in the direction of the sound source. The reaction time towards auditory stimuli, assessed by head turns, has been studied earlier from birth in full-term and premature neonates to 7 months of age (review by Muir & Hains, 2004). The proportion of correct head-turns towards auditory targets in neonates is high but with rather long latency (typically 5–8 s). The development then follows a U-shaped function where latency first increases, and the proportion of correct head-turns decreases, until the age of 2–3 months when the sound lateralization ability starts to improve, and reaction times decrease (1–2 s at 7 months). This U-shaped development process is known from other modalities and tasks, e.g. the neonatal stepping reflex (Gershkoff-Stowe & Thelen, 2004), some areas of language acquisition (Pauls et al., 2013) and theory of mind (Blijd-Hoogewys & van Geert, 2016). There is no satisfying universal explanation to non-linearities in development, but temporary regresses in development might be explained by relapses in neural organization before consolidation of acquired abilities (Blijd-Hoogewys & van Geert, 2016). Head and eye movements in infancy could also be expressions of a sub-cortical reflex that disappears while the cortical interactions are established. Indeed, cortical activity emerges in 2-month-old infants as measured by even-related potentials, however, with longer latencies and lower amplitudes than in older age groups (Slugocki & Trainor, 2014).

Subsequently, sound localization accuracy develops systematically as a function of age between 5 and 18 months of age as shown by increasing acuity in head orientation to sound (Ashmead *et al*., 1991; Morrongiello, 1988; Morrongiello & Rocca, 1987). By age 5 to 6 years, localization accuracy approaches adult performance (van Deun *et al*., 2009), whereas minimum audible angles (i.e discrimination thresholds for the angular difference between two sound sources (Mills, 1958)) continue to decrease into adolescence (Kühnle et al., 2013).

In children with normal hearing, hearing aids, or hearing implants, localization accuracy or acuity has been measured previously by, for example, using the same eye tracking system as in the current study (Asp et al., 2016; Eklöf & Tideholm, 2018; Johansson et al., 2019), pointing/reaching towards or naming the sound source (Asp et al., 2015; van Deun *et al*., 2009; Litovsky et al., 2013), measuring head angle with IR-cameras (Vogt et al., 2018), or by observer-based methods in forced-choice tasks (Grieco-Calub et al., 2008).

The visual system has been optimized for fixation by the fovea where focus changes by saccadic eye movements during which vision is suppressed (Carpenter & Williams, 1995). The magnitude of the saccadic reaction time, and its variability, cannot be explained by simple conduction time, namely synaptic delays and conduction velocity. Instead, the process of reaching the threshold of decision is stochastic in nature and the variability in latency reflect changes in processing times rather than changes in afferent or efferent conduction time (Carpenter & Williams, 1995).

The time from external events in general to a corresponding action varies from reflexes of 7 ms, as with the corneal reflex, to several seconds, as with cognitively demanding response tasks. An example of auditory saccadic reaction is the mediation of gaze during unfolding language. The subject is watching images while the saccadic behavior is mediated by unfolding language, i.e. expressions that instruct the subject what to look at. With eye tracking it is possible to measure the rapid interplay between the earliest phonological moments in the instruction and the corresponding oculomotor control. The minimum saccadic reaction time was found to be 100 ms (Altmann, 2011). The rapid change in direction of one's gaze towards auditory and/or visual targets is called attentional gaze shifts, and can be completely involuntary, as described with visual targets (e.g. the “Capture effect” (Theeuwes et al., 1998)) or voluntarily suppressed depending on context (Hinde et al., 2017). These reactive saccades can be modulated by cognitive areas as shown by the effect of instructions on saccadic response latency (Mosimann et al., 2004). Both saccadic latency (Fischer *et al*., 1993) and accuracy (Kowler & Blaser, 1995) can be improved voluntarily. Furthermore, saccadic reaction does not seem to show an accuracy/latency trade-off; neither accuracy to visual targets (Wu et al., 2010) nor auditory targets (Eklöf et al., 2020) is increased by prolonged motor planning, an otherwise common phenomenon called Fitt's law (Fitts, 1966).

Findlay and Walker (1999) have proposed a model of saccadic processing with two parallel processes: the WHEN process, which produces saccade initiation (i.e. latency) and the WHERE process, which determines saccadic amplitude and direction (i.e. accuracy). The WHEN process can, in turn, be divided into two competing processes. The first process works by upholding fixation, despite changes in the spatial position of attention or emerging, competing visual or auditory targets. The second process works by disentangling fixation and eliciting a saccade. This theoretical model is partly supported by the existence of corresponding brain structures (Munoz & Wurtz, 1995).

The reaction time of gaze towards visual targets, and its development with age, has been investigated. Studies have agreed that saccadic latency to visual targets decreases exponentially from birth to approximately 14–15 years of age when it reaches adult performance (Luna et al., 2008). In infants, reaction time decreases from more than 500 ms, depending on eccentricity, at 1 month of age, to approximately 280 ms at 5 months of age (Regal et al., 1983). An interesting application of how analysis of this development can be used is the prognostic diagnostics of dyslexia during early school age. Eye tracking analysis of saccadic behavior, both its saccadic amplitude and temporal features, during reading can predict future diagnosis with a 95% sensitivity and specificity (Nilsson Benfatto et al., 2016).

Eye-saccadic reaction time towards auditory targets has not been investigated to the same extent. For example, most studies of auditory saccadic performance to date have focused on adults with different studies showing means of e.g. 280 ms (Eklöf et al., 2020), 250 ms (Zambarbieri, 2002), or 190 ms (Zahn et al., 1978) depending on measurement method. A study by Bucci and Seassau (2012) investigated the simple saccadic response latency towards auditory targets from 6 to 15 years of age, but auditory elicited saccadic gaze latencies and their development in infants and young children are to our knowledge unknown. In this study we defined gaze as the combination of eye and head movements when applicable.

While many appropriate methods for the assessment of binaural hearing exist for adults (e.g. Stecker & Gallun, 2012), they are largely unavailable for a young, pre-verbal, population. The aim here was to study whether auditory elicited saccadic gaze latencies could be measured between 0.5 years and 5 years of age and to relate latency to age. We expected the latency to decrease as a function of age.

## Subjects and Methods

### Subjects

The 22 participants (12 females) ranged in age from 0.55 to 5.6 years (mean (SD) = 1.6 (1.4) years). They were all born full term and passed the universal newborn hearing screening (otoacoustic emissions, for details see Berninger and Westling (2011) and Berninger (2014)).

### Study Design

Sound localization latency (SLL) was estimated objectively in infants and young children with normal hearing by fitting a sigmoid function to resemble gaze responses as a function of time during azimuthal sound shifts in the frontal horizontal plane, using the same equipment, auditory-visual stimulus, procedure, and sigmoid model as reported in Eklöf et al. (2020). An inverse regression model was used to study the effect of age on SLL, see the Statistical analysis section below for details.

Sound localization accuracy for twelve of the subjects has been published in another study (Asp et al., 2016), however SLL of the data has not been considered before. Written confirmation for reuse of data in the current study was obtained from the publisher. The study was approved by the regional ethical review board in Stockholm, Sweden (EPN 2012/189–31/3, 2013/2248–32). Written informed consent from accompanying caregiver(s) were obtained for all the subjects and the research complied to the ethical principles of the declaration of Helsinki.

### Sound Localization Test

#### Setup

The setup is described in detail elsewhere (Asp et al., 2016). Briefly, the setup consisted of a custom-made auditory-visual stimulus system and an eye tracking system (Smart Eye Pro, Smart Eye AB, Gothenburg, Sweden).

The auditory-visual stimulus system comprised 12 loudspeakers positioned in 10° increments in a 110-degree arc with 1.2-meter radius, with a 7” TFT-display mounted below each loudspeaker (loudspeaker/display-pairs; LD-pairs). A personal computer (Dell Latitude E5520, Dell Inc, TX) routed sound to the speaker-array by means of a multichannel external soundcard (AudioFire 12, Echo Audio Corporation, CA) and the video by two external multichannel video mixers (VP-108, Kramer Electronics, Israel). A custom-made software programed in MatLab (The Mathworks, Inc, MA) handled the presentation of auditory and visual stimuli and collected gaze data from the eye tracking system.

Subjects were seated in a chair supporting an upright position, or in a caregiver's lap if not accepting to sit by themselves, 1.2 meters from the LD-pairs. The loudspeakers were vertically adjusted to ear-level.

The three-dimensional coordinates of each LD-pair was defined in the eye tracking system as an Area of Interest (AOI) (Asp et al., 2016). Objective detection of eye gaze intersections with the AOIs were performed at 20 Hz. Each sample contained a gaze intersected AOI (i.e. LD-pair 1 to 12) and a time stamp, and was sent from the eye tracking system to the presentation system by a low latency network connection for off-line synchronization (Eklöf et al., 2020). The samples were stored in text files and a compiled dataset can be found at https://doi.org/10.6084/m9.figshare.12311357.v2.

#### Stimulus and Test Procedure

An ongoing auditory-visual stimulus (a colorful cartoon movie with an accompanying melody) was presented starting at −5 degrees azimuth at 63 dB SPL (A), as measured at the position of the subject's head. The auditory stimulus was filtered to resemble the long-term frequency spectrum of speech. The filter was designed to generate the same energy in 1/3-octave bands as the unmodulated noise in the Hagerman sentence recognition test (Hagerman, 1982).

A test consisted of 24 azimuthal sound shifts (trials). The sound was shifted from the loudspeaker in the current LD-pair to another randomly assigned loudspeaker (target) on average every 7th second (range 5 to 9 s). A test lasted for about 3 min if the subject managed to participate in the full set of 24 trials. Off/On-ramping of the sound stimulus at azimuthal shifts were accomplished by a raised cosine of 50 ms. The visual stimulus stopped 170 ms before the azimuthal sound shift and was reintroduced on the visual display corresponding to the sounding loudspeaker 1.6 s after the azimuthal sound shift. The procedure allowed acquisition of gaze behavior in response to a spatial change of the sound. Subjects were free to move their head, but head direction was not recorded. The eye gaze angle was considered relative the room coordinates irrespective of the head angle. Further details are described by Eklöf et al. (2020).

### Objective Determination of Sound Localization Latency Using an Arctangent Function

As a model of the gaze movement during an azimuthal sound shift, an arctangent function was fitted to the samples of gaze intersected AOI. The analysis window for each trial was 4.1 s (82 samples) starting 2.5 s (50 samples) before each azimuthal sound shift and ended when the visual stimulus was reintroduced, i.e. 1.6 s (32 samples) after the azimuthal sound shift.

**Figure 1.**
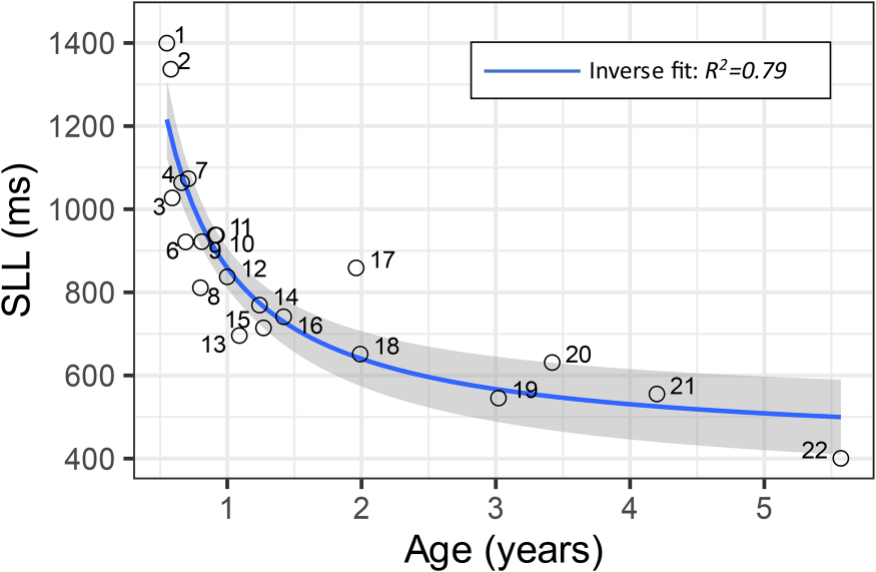
Sound localization latency (SLL) as a function of age for the 21 subjects for which latencies could be obtained. The grey area depicts the 95% confidence interval of the regression.

The time period for the transition of eye gaze from one sound source to another coincides with distinctly increased motor neuron firing rate of the eye muscles (cf. Sparks, 2002, p. 1). Accordingly, the abscissa corresponding to 50% of the amplitude of the fitted arctangent function was defined as the latency T in each trial (Figure 1), i.e. halfway through the localization response. The SLL was defined as the mean of T across trials in a test (*n* ≤ 24 per test). The method is evaluated in adults and described in detail by Eklöf et al. (2020).

#### Sigmoid Model Parameters

The following formula was used to fit an arctangent function to the samples in each trial:

(1) $$a(t)=a_{1}+(a_{2}-a_{1})*\left(\left(\frac{\pi }{2}+\arctan(c*(t-T))\right)/\pi \right)$$

where $a_{1}$ and $a_{2}$ (°) are continuous variables (-55° ≤ $a_{1,2}$ ≤ + 55°) corresponding to the gaze intersected AOI before and after the azimuthal sound shift. The parameter determining the slope *c* (s^−1^) is a measure of the speed when combined with the eccentricity of the trace (0 ≤ *c* ≤ 130), *t* (s) is the time and T (s) is the latency for each trial (T ≥ 0).

#### Trial Exclusion

We applied the strict criteria described and motivated in our previous study in adults (Eklöf et al., 2020). When the analysis window had more than 50% sample loss before or after the loudspeaker shift (due to, for example, insufficient tracking of gaze), the trial was excluded from data analysis. Trials were also excluded when the root mean square error (RMSE) of the arctangent fit was larger than 7° (due to inconsistent eye tracking). Trials with a *T* = 0 and *T* > 1.6 s, i.e. longer than the sound only period, were also excluded.

One additional criterion that was not necessary in the previous study in adults (Eklöf et al., 2020) was needed in the current study; trials where the arctangent fit of $a_{1}$ and $a_{2}$ was below 10 degrees (the smallest separation between two LD-pairs) were excluded since this was indicative of a flat response with no actual gaze shift.

### Statistical Analyses

The statistical software used was R version 3.4.2 (https://www.R-project.org/). Previous studies on development of visually guided saccadic latencies (Luna et al., 2004) suggest that inverse regression models best describe variability in latencies across ages. Hence, inverse regression analysis was performed to study the relationship between SLL and age ( $SLL=b_{0}-b_{1}/Age$ ), where *b*_0_ and *b*_1_ are linear regression parameters. The first parameter, $b_{0}$ , is the asymptote of SLL as age increases. The second parameter, $b_{1}$ , is a measure of the steepness of the inverse decay. This regression was further established by a Boot Strapping procedure (package *boot*) presented in the supplement section.

A Monte Carlo simulation was performed to assess the effect of number of trials on SLL utilizing the *snorm* function of the *fGarth* package to obtain values from a skewed distribution.

A linear mixed model (package *lmer*) was used to study the following fixed effects/factors on latency: angular separation between the previous LD-pair and the target LD-pair, azimuth after shift, trial number in the test, and subject sex. Subject was included as random factor since trials within a subject were not independent.

Sound localization accuracy was quantified by an Error index (EI), a normalized mean absolute error where 1.0 corresponds to random performance and 0.0 is perfect localization. The responded loudspeaker was defined as the median gaze intersection from the last 500 ms samples of the sound-only period (Asp et al., 2016). The EI is calculated by the following formula: $EI=\frac{\sum_{i=1}^{n}\;|\;p_{i}-r_{i}|}{\left(\sum_{\;j=1}^{n}\;\sum_{k=1}^{m}\;|\;p_{j}-q_{k}|\right)/n}$ , where the nominator accumulates the total absolute error across *n* presentations *p* with valid responses *r* and the denominator accumulates the error of all *m* possible responses *q* of the available response space averaged across each of *n* presentations. A Monte Carlo simulation by Asp et al. (2016) showed that EI = 0.72 is the lower limit of the 95% confidence interval for random performance in this setup.

Partial correlations between EI and SLL and corresponding significant levels were calculated using the *psych* package, and repeated measures correlation with the *rmcorr* package.

## Results

### Sound Localization Latency Decreases as a Function of age

The overall mean (SD) SLL was 860 (260) ms (*n* = 21 subjects) with a range of 400–1400 ms. The SLL was found to vary with age. Inverse regression analysis revealed a distinct and statistically significant effect of age on SLL ( SLL=416+455/Age , *R*^2^ = 0.79, *p* < 0.001, Figure 1). The decrease in SLL was most pronounced before ≈ 1.5 years of age, after which the SLL seemed to decrease slowly towards approximately 500 ms (Figure 1). The distribution of the participants’ ages was normally distributed on an inverse scale and the inverse regression was confirmed by a Boot Strapping procedure (95% confidence interval [320, 530 ms/year^−1^] with 10000 replicates, Figure S1).

### Fitting of Arctangent Function

**Figure 2.**
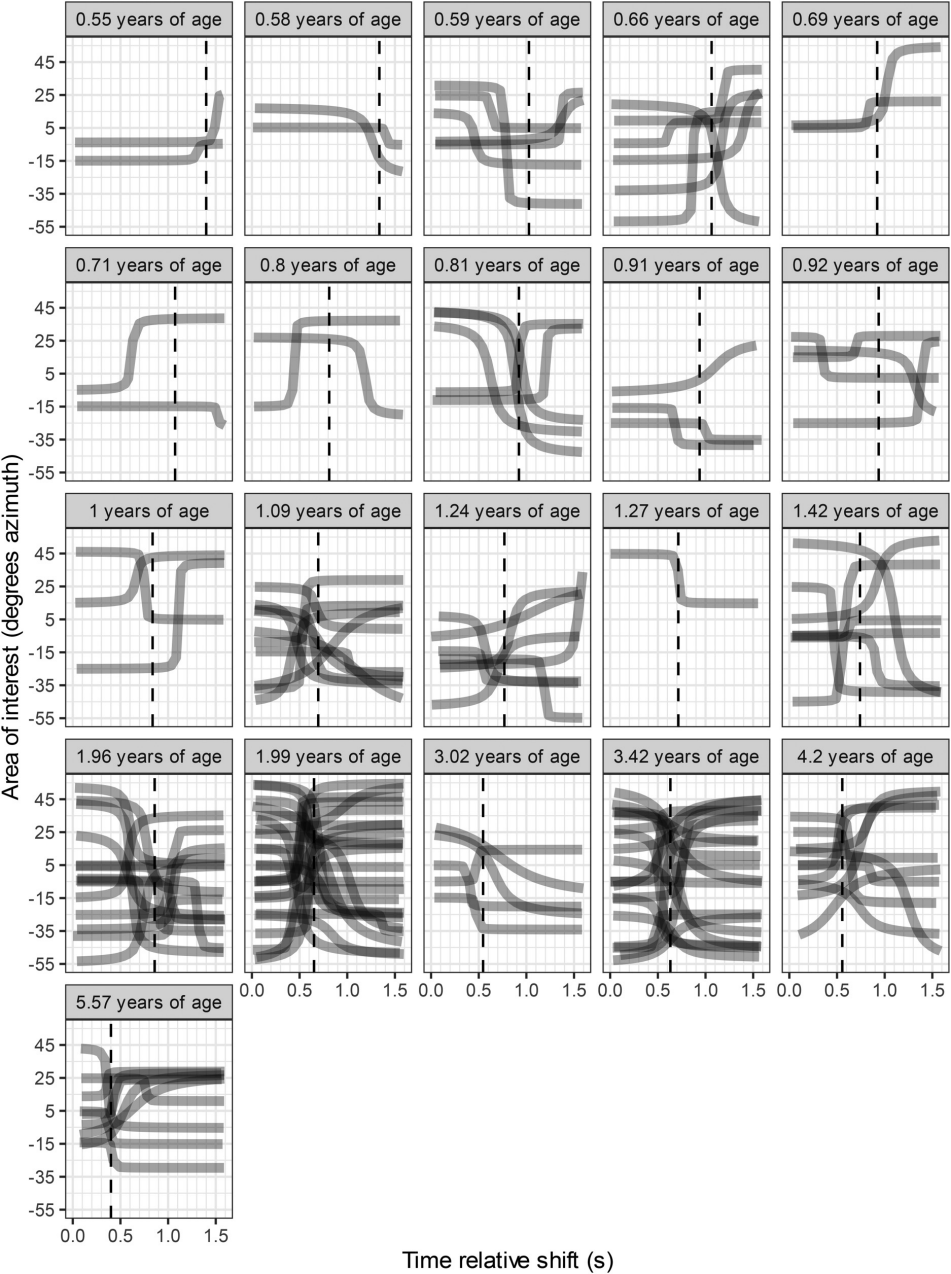
Included sigmoid functions, based on samples of intersections between eye gaze and areas of interest, for each subject. The heading of each panel denotes the subject's age in years. The time axis includes the auditory-only period of 1.6 s. The vertical dashed line indicates the SLL for the subject. The subject was fixating on the previous target LD-pair which presented both auditory and visual stimuli before the next trial in accordance with the current procedure.

Figure 2 shows the fitted sigmoid models for included trials for each subject in ascending age.

The mean proportion of trials that passed the inclusion criteria for calculation of latency increased with age (*R*^2^ = 0.52, *p* < 0.001). Due to the limited number of fitted trials in children <1 years of age, a Monte Carlo simulation was performed to study the validity of the age dependency of the SLL measure. The simulation hypothesized that the latencies originated from the same distribution. The distribution used for simulation resembled the distribution of latencies for adults (Eklöf et al., 2020) which was a normal distribution but with a skewness towards lower values, ξ = 2. A conservative standard deviation of 300 ms was chosen after considering that subjects with 5 fitted trials or more exhibited a mean standard deviation of 230 ms (Table S1). The Monte Carlo simulation revealed that a slope parameter of 415 ms/years^−1^ or more was very unlikely (*p* < 0.0001, 10^5^ simulations), see histogram of the simulated slopes in Figure S2.

Figure S3 in the supplements shows all trials for all children. Gaze intersected AOI and corresponding arctangent functions are depicted as well as the trial latency. The youngest subjects showed a gaze behavior that was more often automatically excluded by the objective criteria, as seen in Table S1 in the supplements. Visual inspection confirms the validity of most of these exclusions, however the objective criteria occasionally included or excluded trials that may had been evaluated differently by visual inspection. As an example, one of the excluded trials (trial 6) for subject 1 had a distinct gaze shift in the sound-only period but the gaze shift immediately before the start of the sound-only period resulted in exclusion by the objective criteria. As another example, for subject 7 (age = 0.8 years), the objective criteria included trial 14 although visual inspection indicated that gaze patterns before and during the sound-only period were unstable (Figure S3).

### The Effects of Separation Between Loudspeakers, Azimuth After Shift, Time Elapsed, and sex on Latency

Latencies for all included trials varied from 270–1500 ms (*n* = 128 trials in 21 subjects). This variability was studied with a linear mixed model with the effects of angular separation between loudspeakers, the azimuth after a shift, and the time elapsed during a test, on SLL. Backward reduction with Satterthwaite's method for calculation of degrees of freedom resulted in no significant fixed effect (azimuth after shift, *p* = 0.8; sex, *p* = 0.4; separation between loudspeakers, *p* = 0.3; time elapsed, *p* = 0.1).

### No Relationship Between Sound Localization Latency and Accuracy When Controlling for age

**Figure 3.**
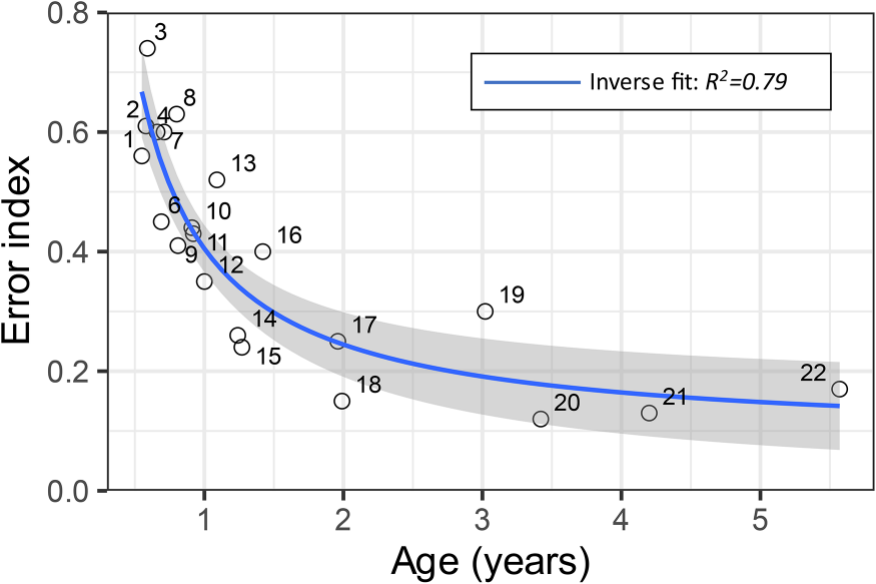
Sound localization accuracy (Error index) for all 21 subjects. The grey area depicts the 95% confidence interval of the regression. The accuracy (Error index) of 12 of the subjects was presented in Asp et al. (2016).

In addition to the decrease in SLL there was a decrease in EI (i.e. an increase in accuracy) as a function of age ( EI=0.08+0.3/Age , *p* < 0.0001, *R*^2^ = 0.79, *n* = 22) (Figure 3).

**Figure 4.**
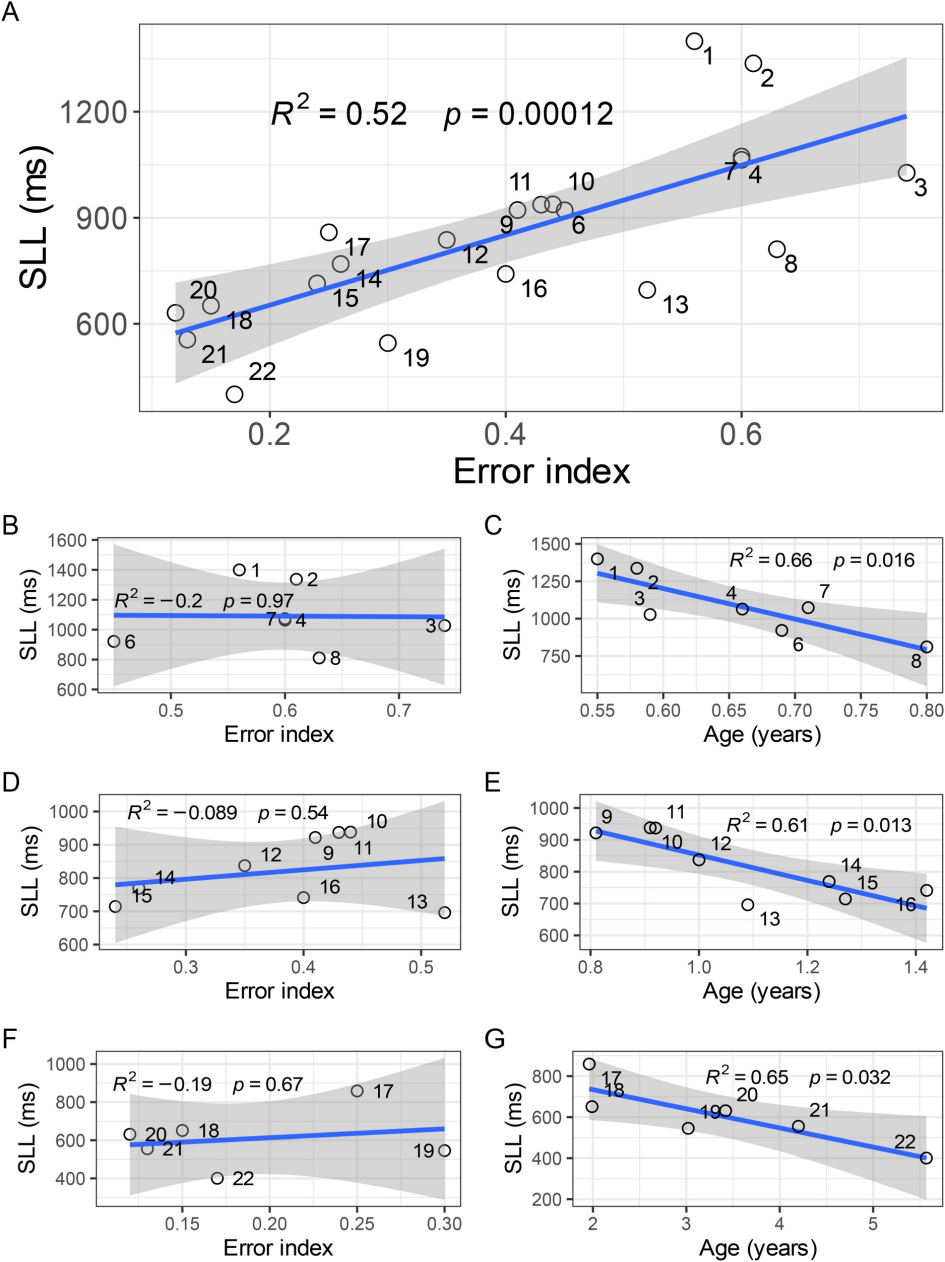
The grey areas depict the 95% confidence interval of the linear regressions. While A shows SLL as a function of age for all subjects (*N* = 21), B, D, and F shows the same analysis but divided in three age groups with 6-8 subjects in each age interval. C, E, G shows the corresponding linear regression between SLL and EI in the same age intervals (B, C:  ≤ 0.8 years, *N* = 7, D, E: >0.8 and  ≤ 1.6 years, *N* = 8, F, G: >1.6 years of age, *N* = 6)). Notably, the correlation of SLL and age is significant in each age group whereas there is no correlation between SLL and EI. The accuracy (Error index) of 12 of the subjects was presented in Asp et al. (2016).

Accordingly, age had a distinct influence on both SLL and EI. By this relationship, there was also a correlation between SLL and EI, Figure 4A. To study whether SLL was related to EI when controlling for the effect of age, we divided the subjects in three age groups and calculated the correlation between SLL and EI (panels B, D, and F in Figure 4) and the correlation between SLL and age (panels C, E, and G in Figure 4). It follows from these analyses that there was no relationship between SLL and EI whereas the correlation between SLL and age remained. Additionally, we examined the relationship between accuracy and latency using partial correlation while controlling for age. No partial correlation existed between the SLL and EI (*R_partial_* *=* *–* 0.19, *p* = 0.51, *N* = 21).

In addition, no correlation between the latency and the corresponding absolute localization error existed in each trial (i.e. the absolute difference in degrees between the target azimuth and the perceived azimuth) (*R*^2^ = 0.007, *p* = 0.4, repeated measures correlation).

## Discussion

The latency of gaze shifts toward auditory targets in the frontal horizontal plane was measured in infants and young children between 0.5 and 5.6 years of age. Latencies showed high variability and a distinct age-dependence, such that SLL decreases with increasing age. Accuracy of localization responses were also improved as a function of age, but not associated with latencies. The demonstrated age-dependence for latency and accuracy in the studied age range contrasts with those found for visual stimuli (Alahyane et al., 2016). While latencies towards visual stimuli for children aged 5 to 42 months decreased significantly with age, the magnitude of that decrease was much smaller (latencies were 290 ms at 5 months and 200 ms at 42 months) than for the auditory targets in the current study.

We utilized a corneal-reflection eye tracking technology that we previously have used to assess localization accuracy in infants and young children (Asp et al., 2016), in children with unilateral hearing loss (Johansson et al., 2019), and cochlear implant recipients (Eklöf & Tideholm, 2018). The method was then extended and developed to objectively assess localization latency in adults (Eklöf et al., 2020). In the present study, we show that the same method was applicable in children. The on-going audiovisual stimulus with azimuthal shifts, and offline objective modeling of the gaze responses, provided a measure of latency rapidly, without the need of manual evaluation of trials that could potentially introduce subjective bias. Monte Carlo simulation showed that despite a relatively low number of trials included by objective criteria below 12 months of age, a robust age dependency for SLL exists.

Gaze-saccadic behavior is a natural response to external events and tasks, with a lifetime of “training”. Eye tracking allows for detailed recording of saccadic behavior. When used with equipment that provides freedom of head movements, as in the current study, the test situation allows quite natural behavior despite being in the laboratory. Oculomotor tasks are known to follow repeatable patterns of brain activation of large parts of the brain (Carpenter & Williams, 1995). Hence, the many features of saccadic behavior can provide insight into normal and pathologic development of different brain areas and functions, see for example a review by Hutton (2008). The simple saccadic response task in the current study included natural and intuitive responses. This eliminated the need of verbal instructions and made assessment of sound localization behavior in infants and young children possible without prior conditioning.

Sound localization accuracy is thoroughly investigated in children with normal and impaired hearing (Asp et al., 2015, 2016; Ausili et al., 2019; Eklöf & Tideholm, 2018; Grieco-Calub et al., 2008; Litovsky et al., 2013; Nordlund, 1964). The rapid and objective test described here quantified accuracy and latency (e.g. WHERE and the WHEN processes) of horizontal sound localization in the same task. Wickelgren (1977) and Fitts (1966) argue that analysis of behavioral responses should not only consider accuracy but also take latency into account. Accordingly, the accuracy/latency trade-off can hide differences in effort between conditions. This trade-off, however, might not hold true for saccadic response latency as found in studies of both visual targets (Wu et al., 2010) and auditory targets (Eklöf et al., 2020) since there was no increase in accuracy with increased latency. Similar to the decrease in SLL across ages, the accuracy (i.e. EI) was modeled by an inverse fit (*R*^2^ = 0.74, *p* < 0.0001), reflecting increasing accuracy with increasing age. Including adult data (Asp et al., 2016) in the modeling of development of accuracy resulted in a lower asymptote ( EI=0.055+0.34/Age , *R*^2^ = 0.89, *p* < 0.0001, *n* = 26). Similar results were found for the SLL when we included adults with normal hearing (age range: 18–40 years, *n* = 8) from a previous study (Eklöf et al., 2020). The yielded model of the latency resulted in the following formula: SLL=310+540/Age (*R*^2^ = 0.88, *p* < 0.001, *n* = 29).

We found no partial correlation between EI and SLL when controlling for age (*p* = 0.51) which suggests that accuracy and latency follows different trajectories in different individuals. A positive partial correlation would have indicated that subjects with high accuracy also would have responses with short latency. On the other hand, had the partial correlation been negative, it would have indicated that subjects with high accuracy would tend to show longer latencies. The lack of partial correlation indicates that accuracy and latency may reflect different aspects of development. Concludingly, either of the measures can be used to determine typical development in children with normal hearing, whereas one might speculate that certain pathological conditions might be easier to detect using the latency measure. The latency and accuracy, and its relationship, remains to be studied in clinical cohorts.

## Conclusions

Auditory elicited saccadic gaze latencies may be objectively assessed in children between 0.5 and 5 years of age, but latencies are challenging to obtain before 1 years of age. SLL decreases significantly as a function of age, from about 1 s at 6 months of age to about 500 ms at 3 years of age. The decrease is most pronounced before ≈1.5 years of age. The results suggest that SLL can be used as a behavioral measure of binaural processing in infants and young children.

## Supplemental Material

sj-docx-1-tia-10.1177_23312165221088398 - Supplemental material for The Development of Sound Localization Latency in Infants and Young Children with Normal Hearing Click here for additional data file. Supplemental material, sj-docx-1-tia-10.1177_23312165221088398 for The Development of Sound Localization Latency in Infants and Young Children with Normal Hearing by Martin Eklöf, Filip Asp and Erik Berninger in Trends in Hearing

## Abbreviations:

SLL: Sound localization latency
AOI: Area of interest
EI: Error index
LD-pair: Loudspeaker/Display-pair
