# Supplementary material for: The Development of Sound Localization Latency in Infants and Young Children with Normal Hearing
Source: Trends Hear. 2022 May 3;26:23312165221088398. doi: 10.1177/23312165221088398 (PMC9073128; doi:10.1177/23312165221088398)
Supplement: sj-docx-1-tia-10.1177_23312165221088398 - Supplemental material for The Development of Sound Localization Latency in Infants and Young Children with Normal Hearing [file sj-docx-1-tia-10.1177_23312165221088398.docx]

## Supplements online

Table S1

| Subject ID | Age (years) | Number of participated trials | Fitted  Trials | SLL (ms) | Min latency (ms) | Max latency (ms) | IQR (ms) | SD (ms) | EI |
| --- | --- | --- | --- | --- | --- | --- | --- | --- | --- |
| 1* | 0.55 | 15 | 2 | 1400 | 1300 | 1500 | 94 | 130 | 0.56 |
| 2 | 0.58 | 24 | 2 | 1300 | 1300 | 1400 | 71 | 100 | 0.61 |
| 3* | 0.59 | 17 | 6 | 1000 | 460 | 1500 | 710 | 460 | 0.74 |
| 4 | 0.66 | 24 | 65 | 1100 | 620 | 1400 | 250 | 280 | 0.60 |
| 5 | 0.67 | 8 | 0 | - | - | - | - | - | - |
| 6* | 0.69 | 19 | 2 | 920 | 820 | 1000 | 100 | 140 | 0.45 |
| 7 | 0.71 | 24 | 2 | 1100 | 620 | 1500 | 460 | 650 | 0.60 |
| 8* | 0.8 | 16 | 2 | 810 | 440 | 1200 | 370 | 530 | 0.63 |
| 9* | 0.81 | 13 | 5 | 920 | 630 | 1200 | 62 | 210 | 0.41 |
| 10 | 0.91 | 24 | 3 | 940 | 690 | 1100 | 210 | 220 | 0.44 |
| 11 | 0.92 | 24 | 4 | 940 | 350 | 1400 | 730 | 500 | 0.43 |
| 12* | 1 | 24 | 3 | 840 | 660 | 1100 | 220 | 240 | 0.35 |
| 13* | 1.09 | 23 | 10 | 700 | 410 | 1100 | 270 | 230 | 0.52 |
| 14 | 1.24 | 24 | 6 | 770 | 490 | 1100 | 410 | 270 | 0.26 |
| 15* | 1.27 | 9 | 1 | 710 | 710 | 710 | 0 | NA | 0.24 |
| 16* | 1.42 | 24 | 7 | 740 | 480 | 1100 | 340 | 240 | 0.40 |
| 17 | 1.96 | 24 | 14 | 860 | 580 | 1300 | 390 | 230 | 0.25 |
| 18* | 1.99 | 24 | 23 | 650 | 390 | 1400 | 240 | 230 | 0.15 |
| 19* | 3.02 | 10 | 5 | 550 | 400 | 710 | 190 | 140 | 0.30 |
| 20* | 3.42 | 24 | 19 | 630 | 480 | 840 | 130 | 95 | 0.12 |
| 21 | 4.2 | 24 | 9 | 560 | 340 | 740 | 130 | 130 | 0.13 |
| 22 | 5.57 | 24 | 7 | 400 | 270 | 700 | 150 | 160 | 0.17 |

Abbreviations: F=female, M=male, SLL = Sound Localization Latency, IQR = Inter quartile range, SD = Standard deviation, EI = Error index, subjects with an asterisk (*) had their EI-values presented in Asp et al. (2016)


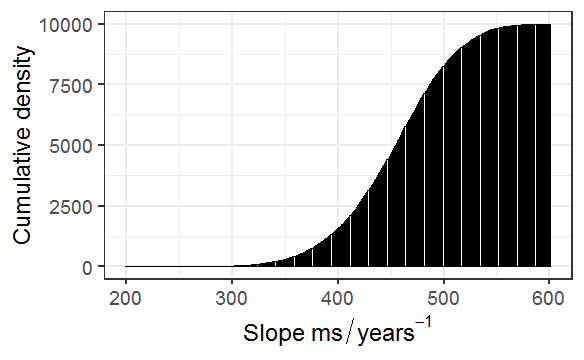


Figure S1. The inverse regression of SLL to age was boot strapped. The figure depicts the cumulative density function of the slope parameter.


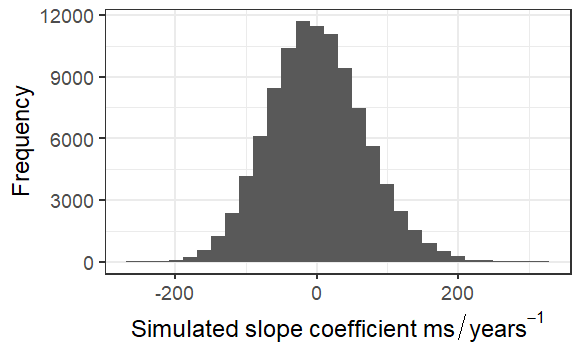


Figure S2. Histogram of inverse regression slopes from a Monte Carlo simulation where SLLs were calculated from the same number of trials as the study data. The simulation was carried out under the assumption that there was no age dependency and thus trial latencies were sampled from the same distribution.


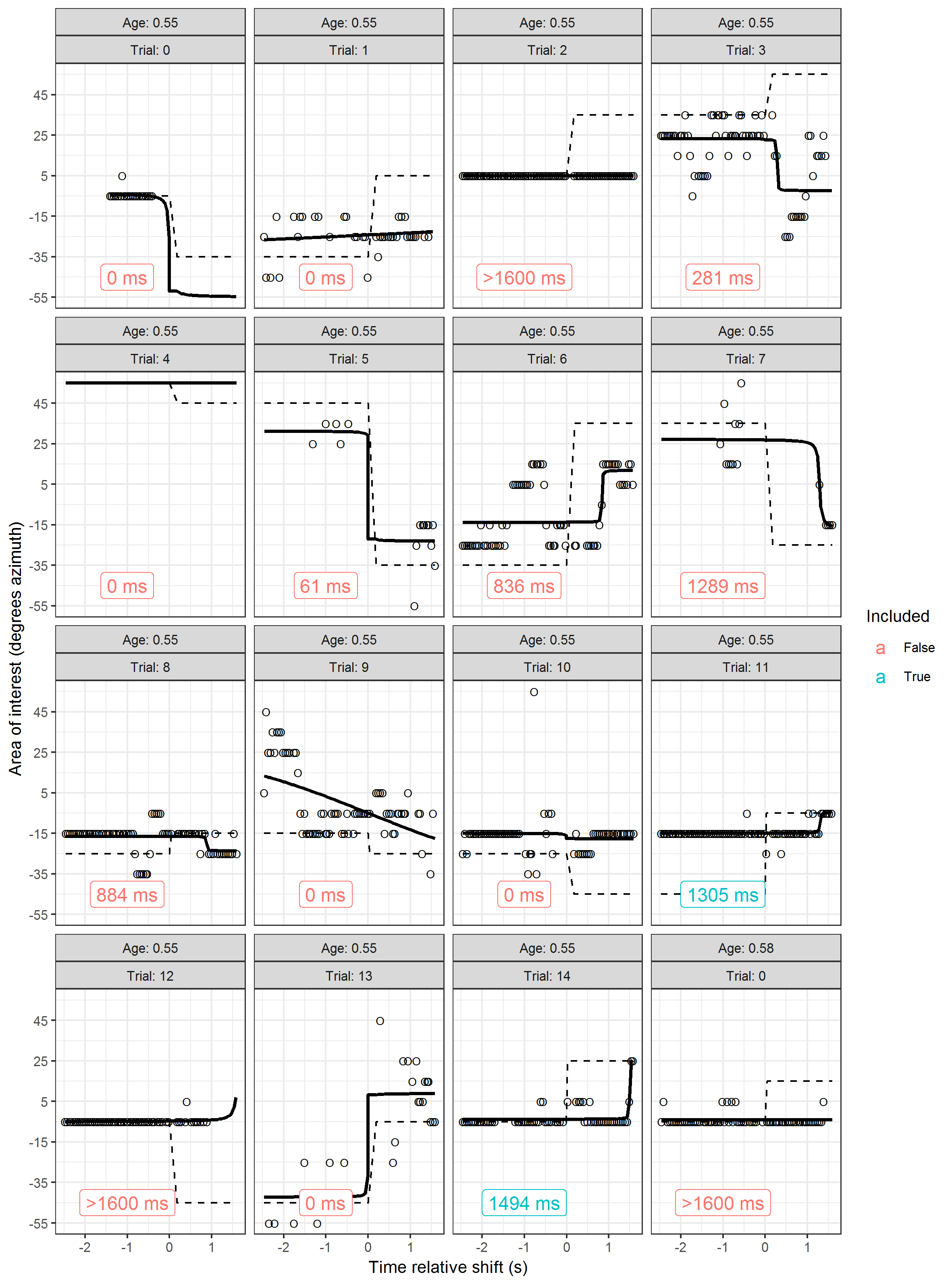

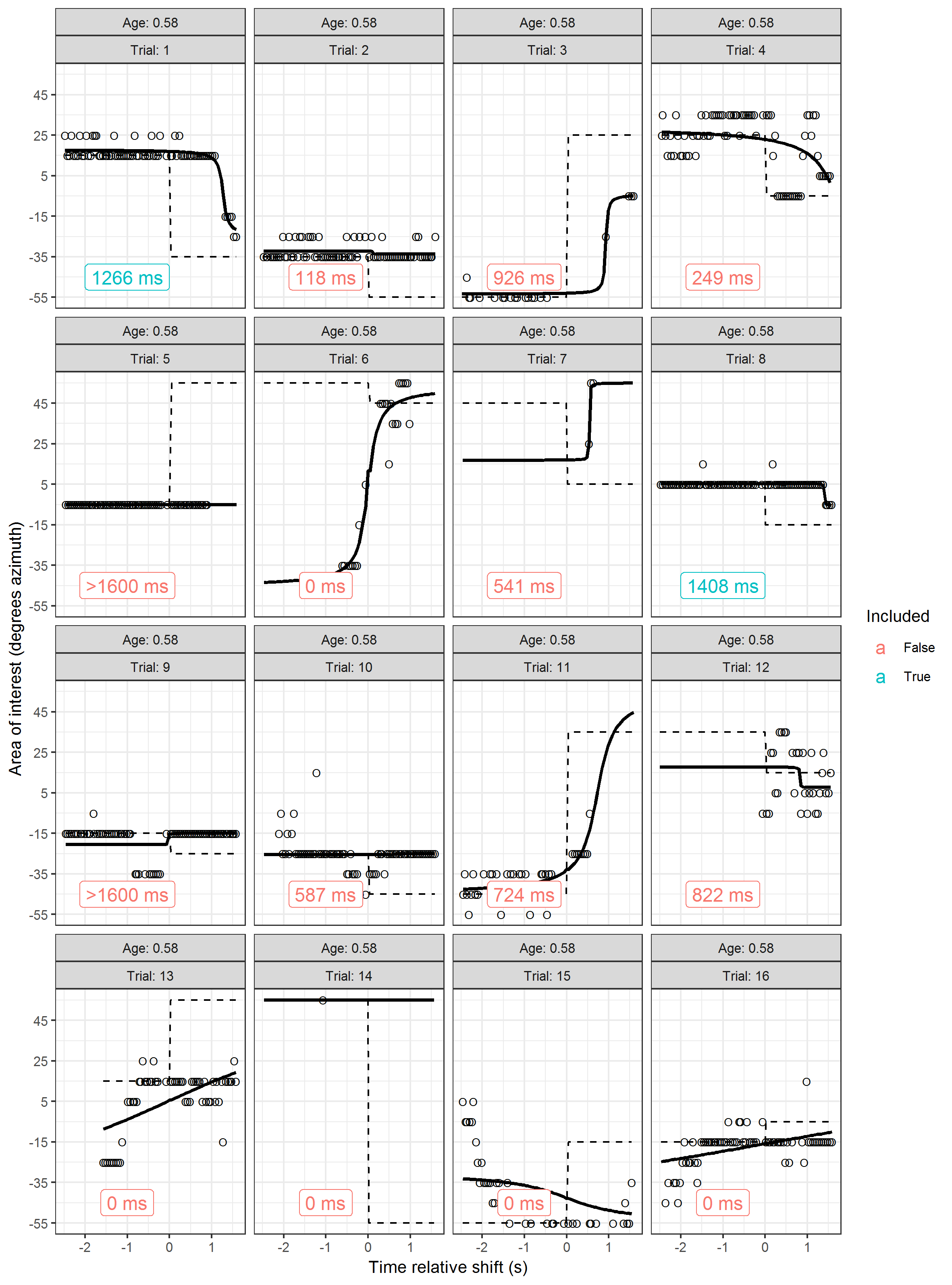

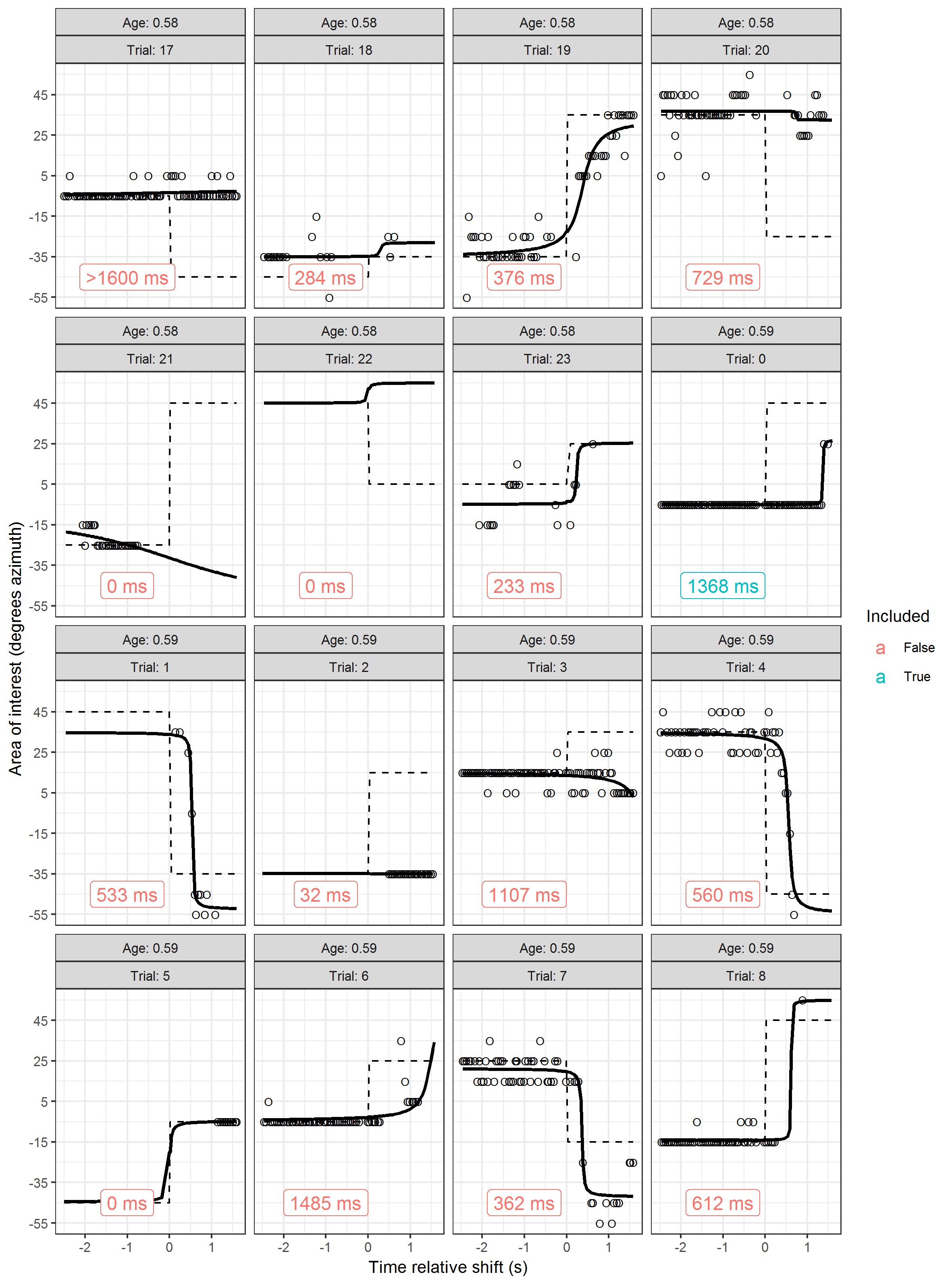

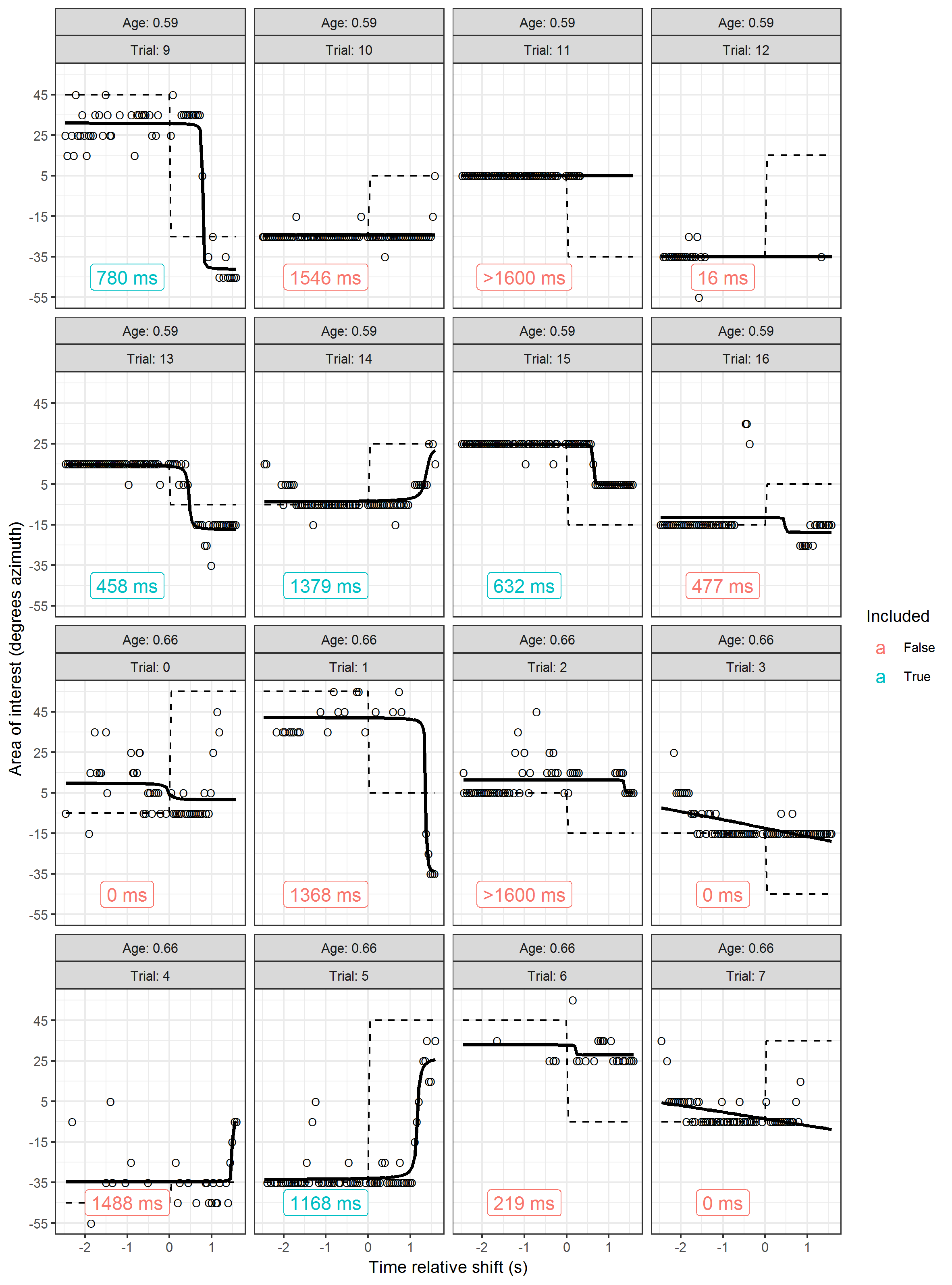

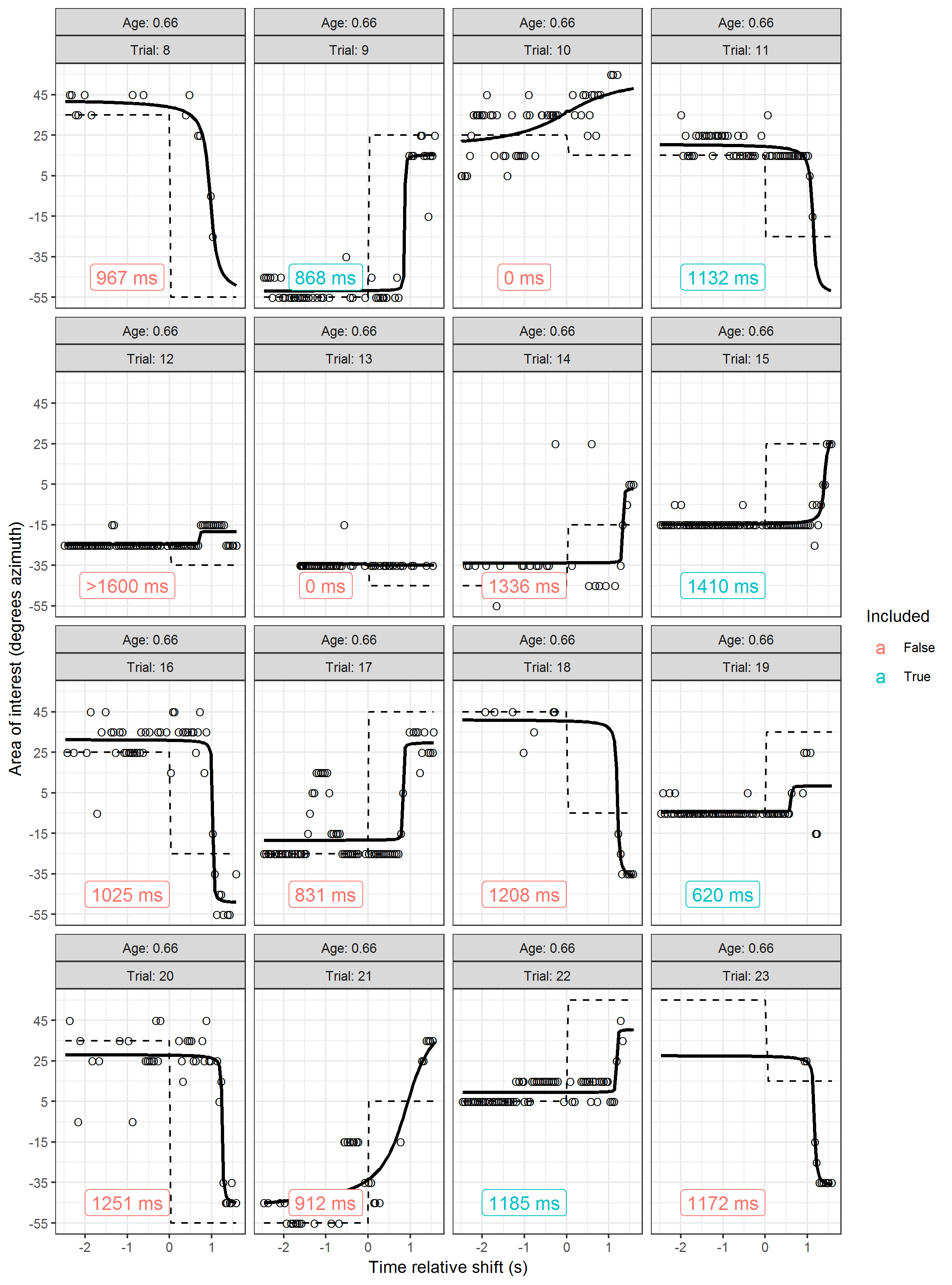

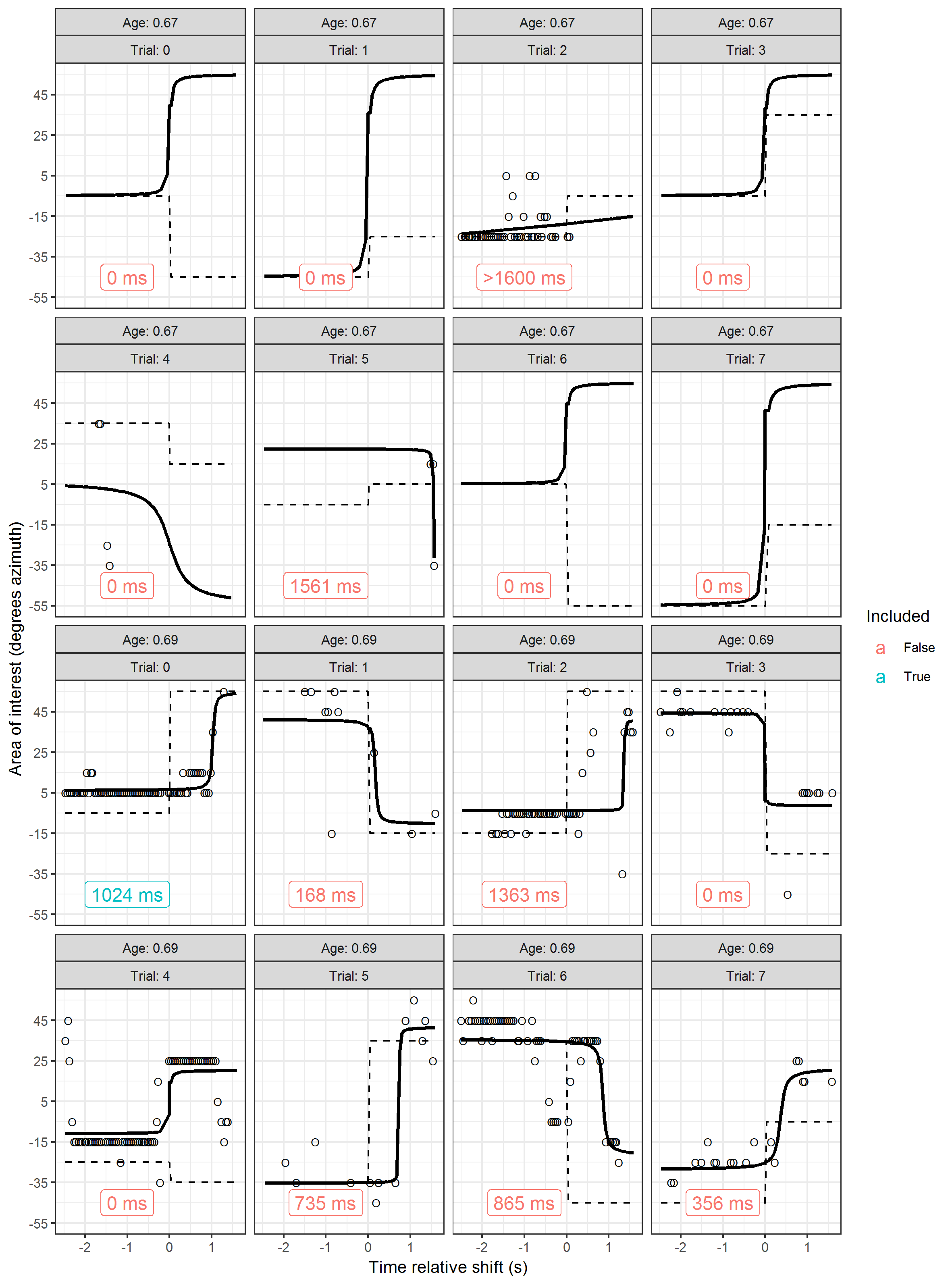

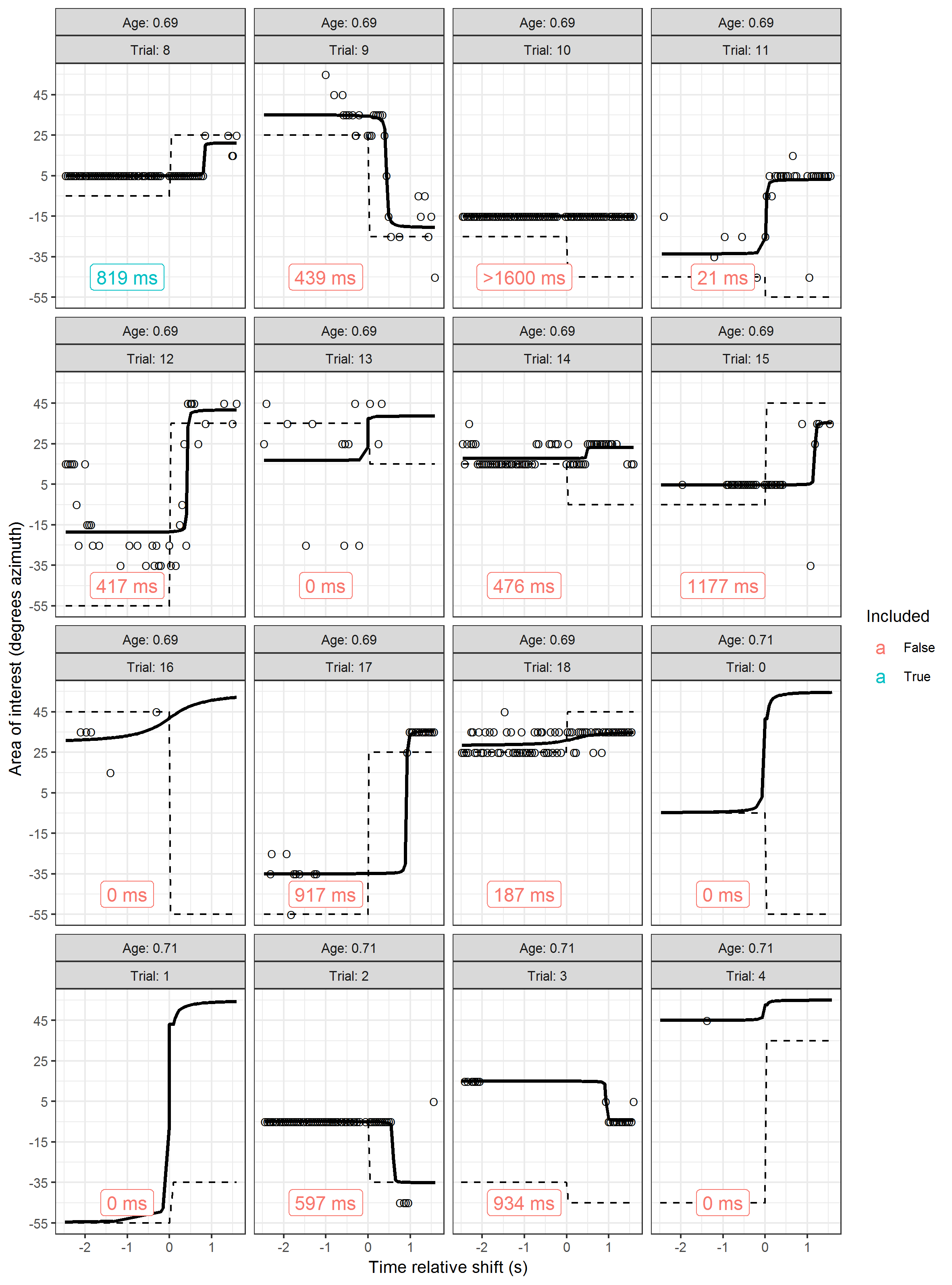

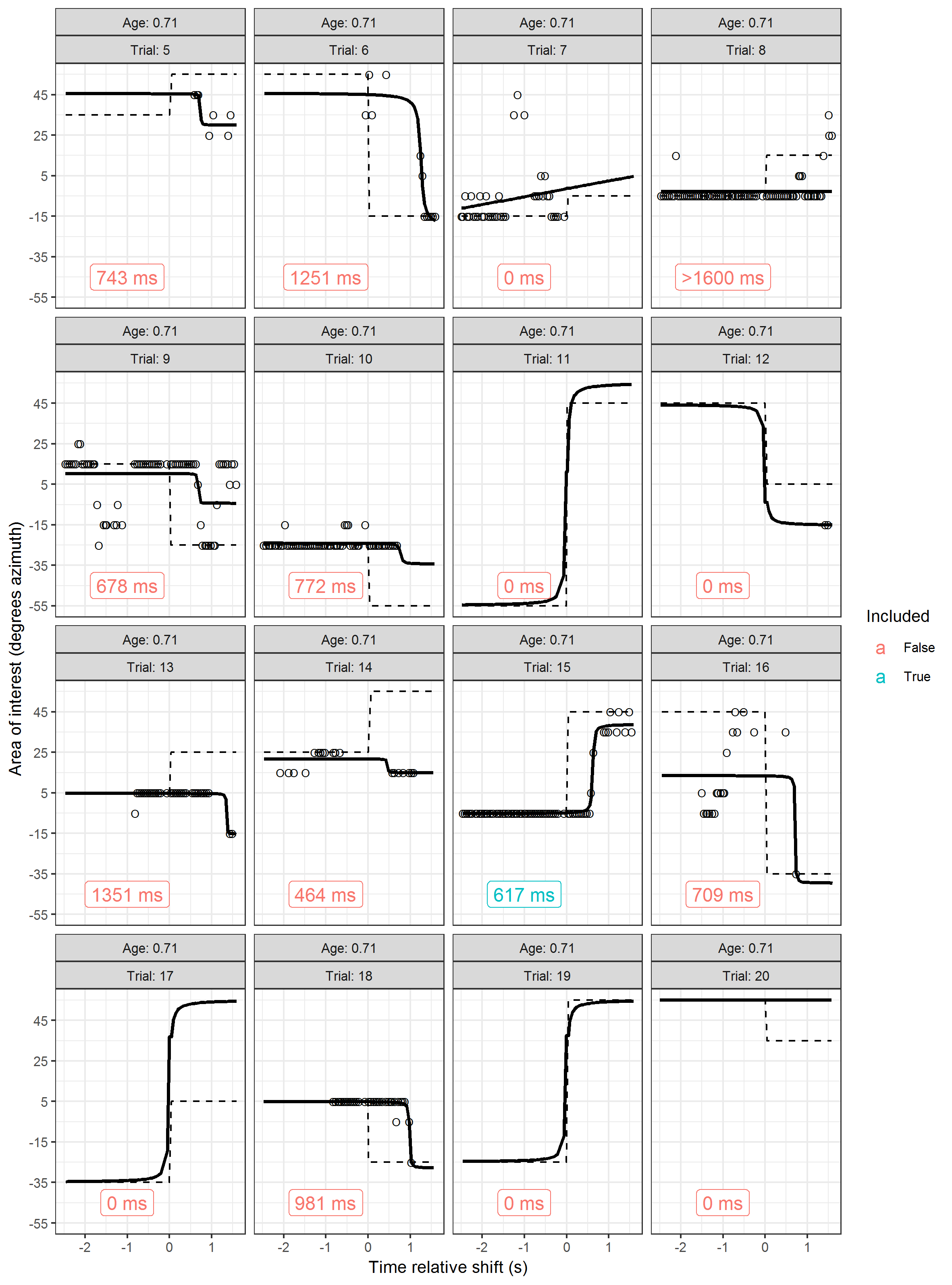

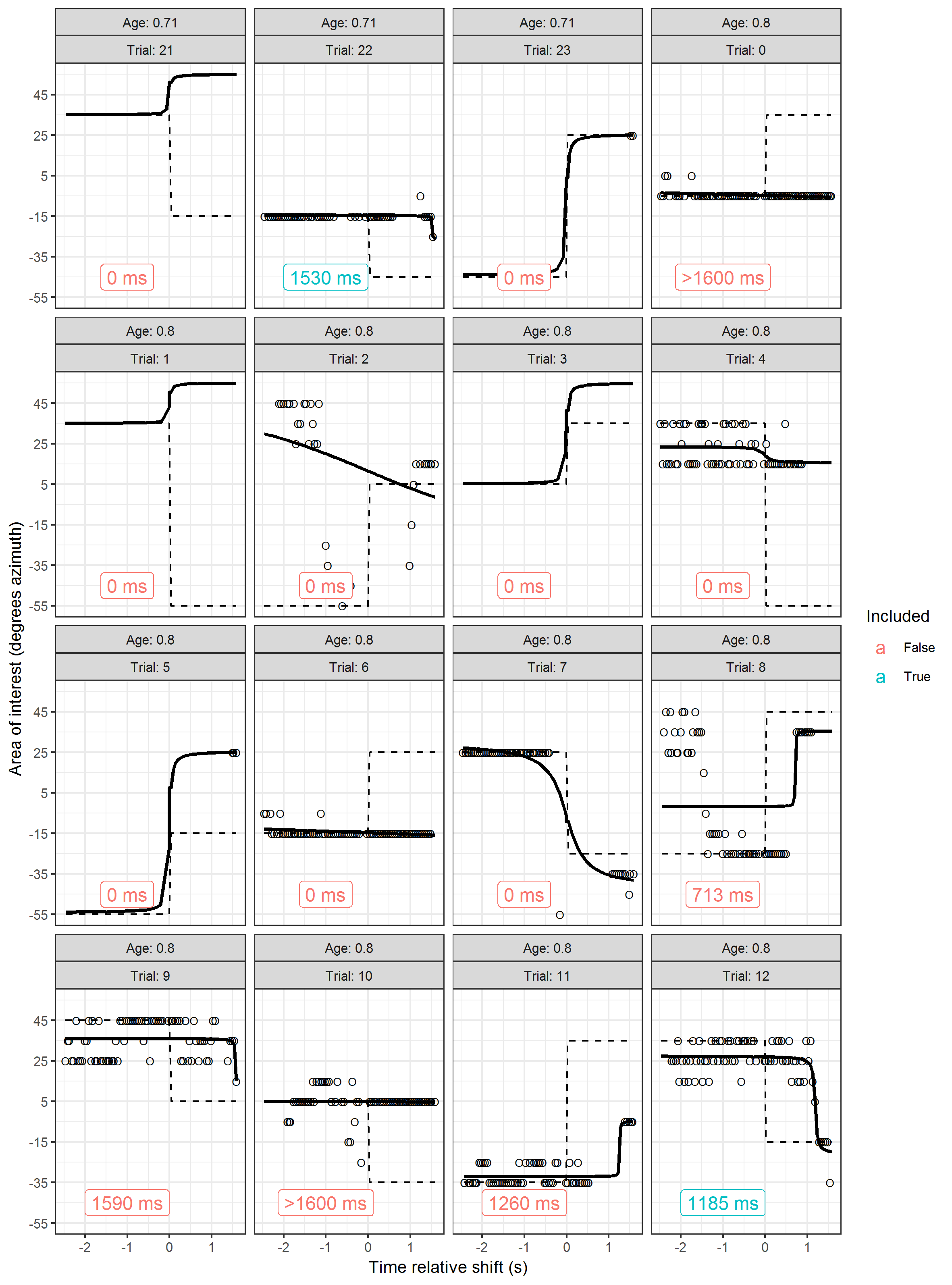

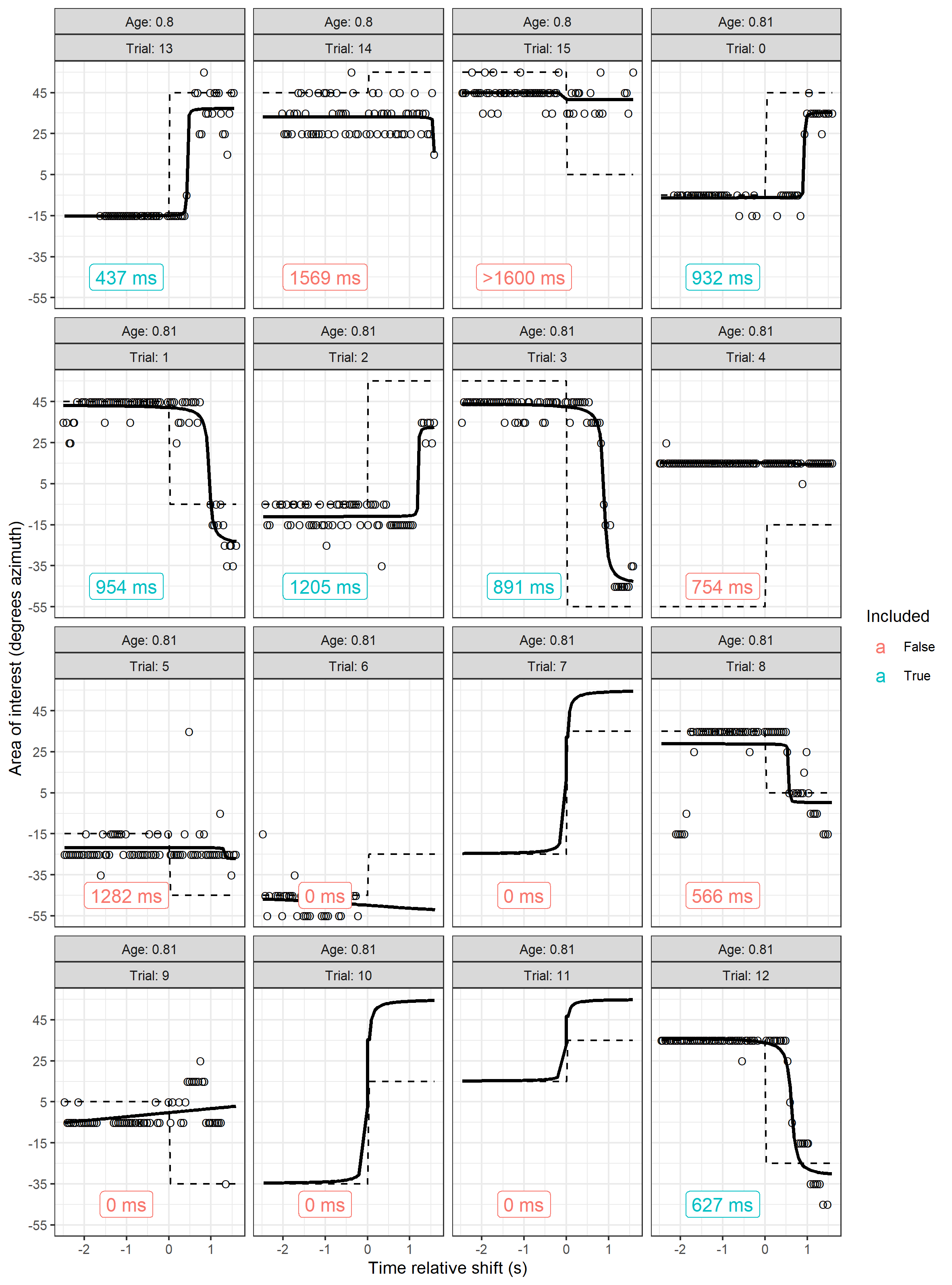

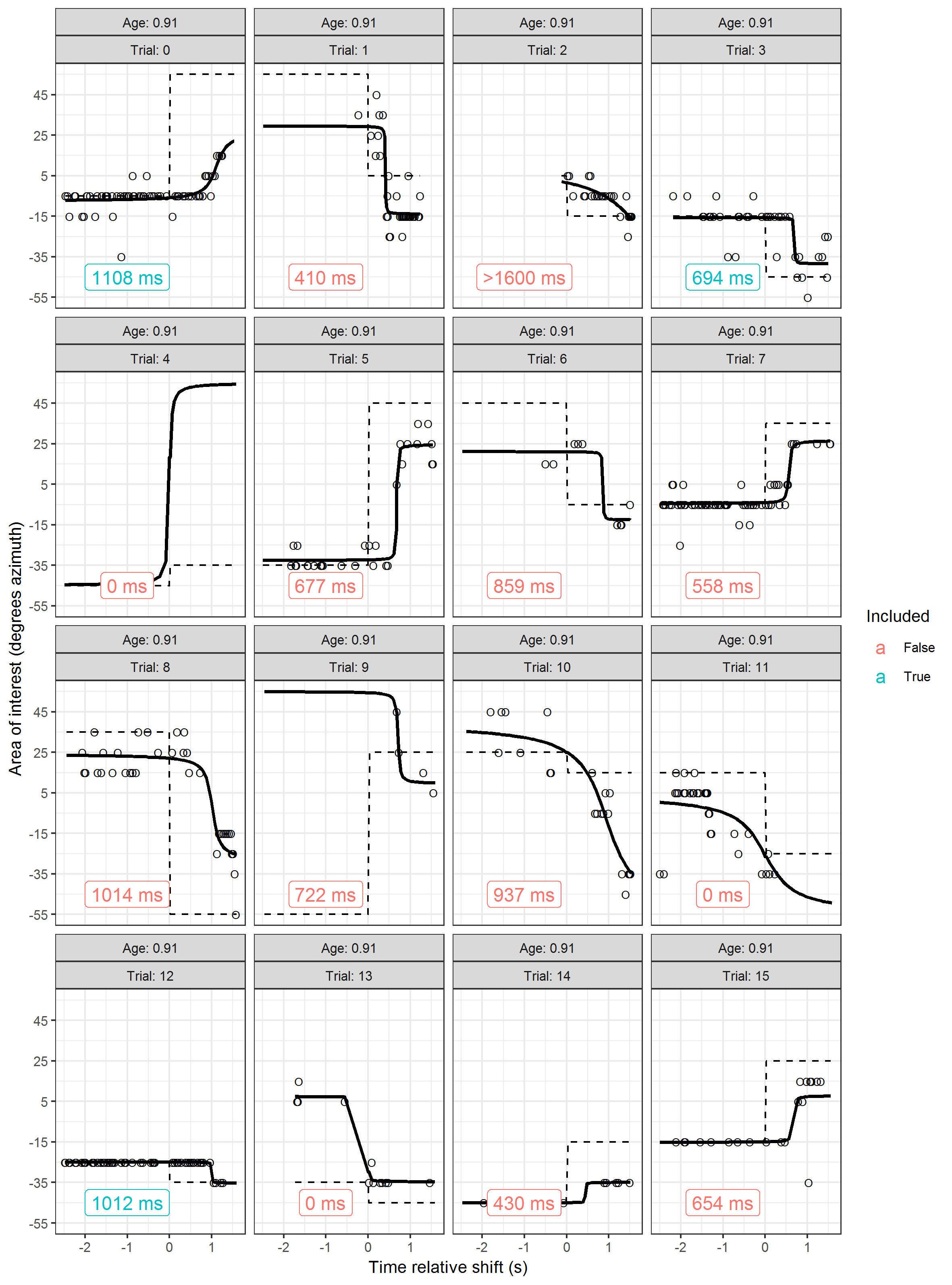

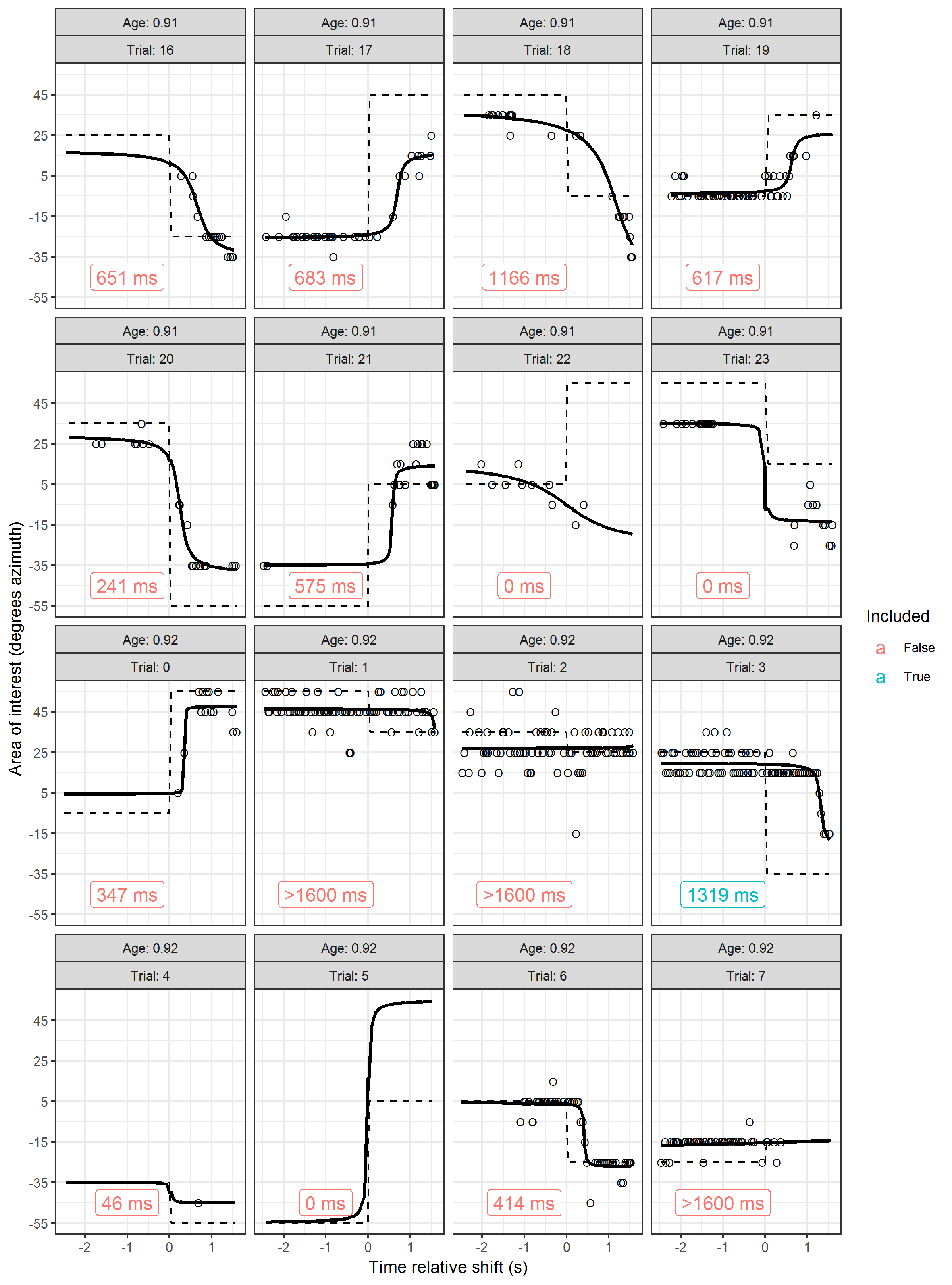

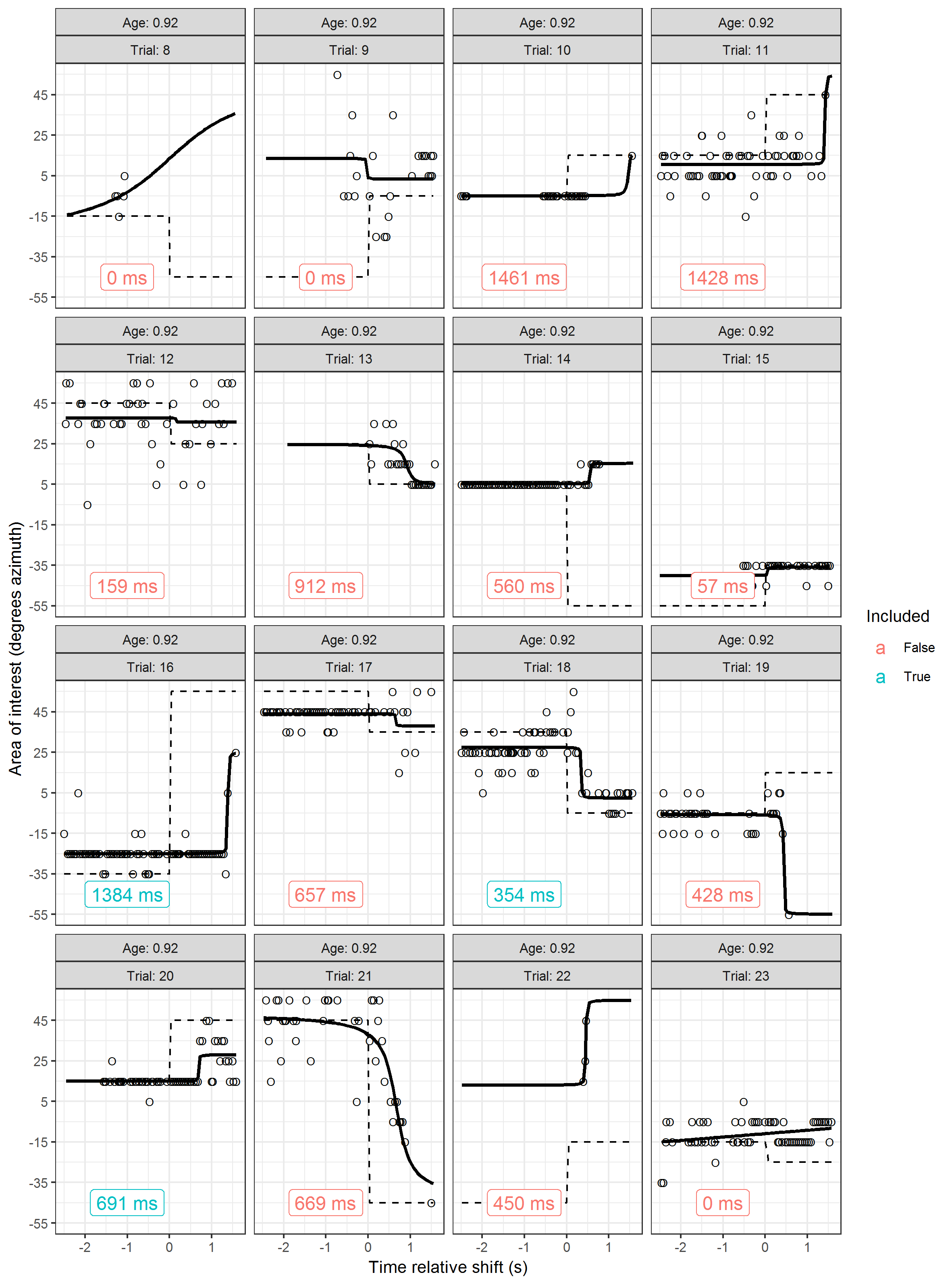

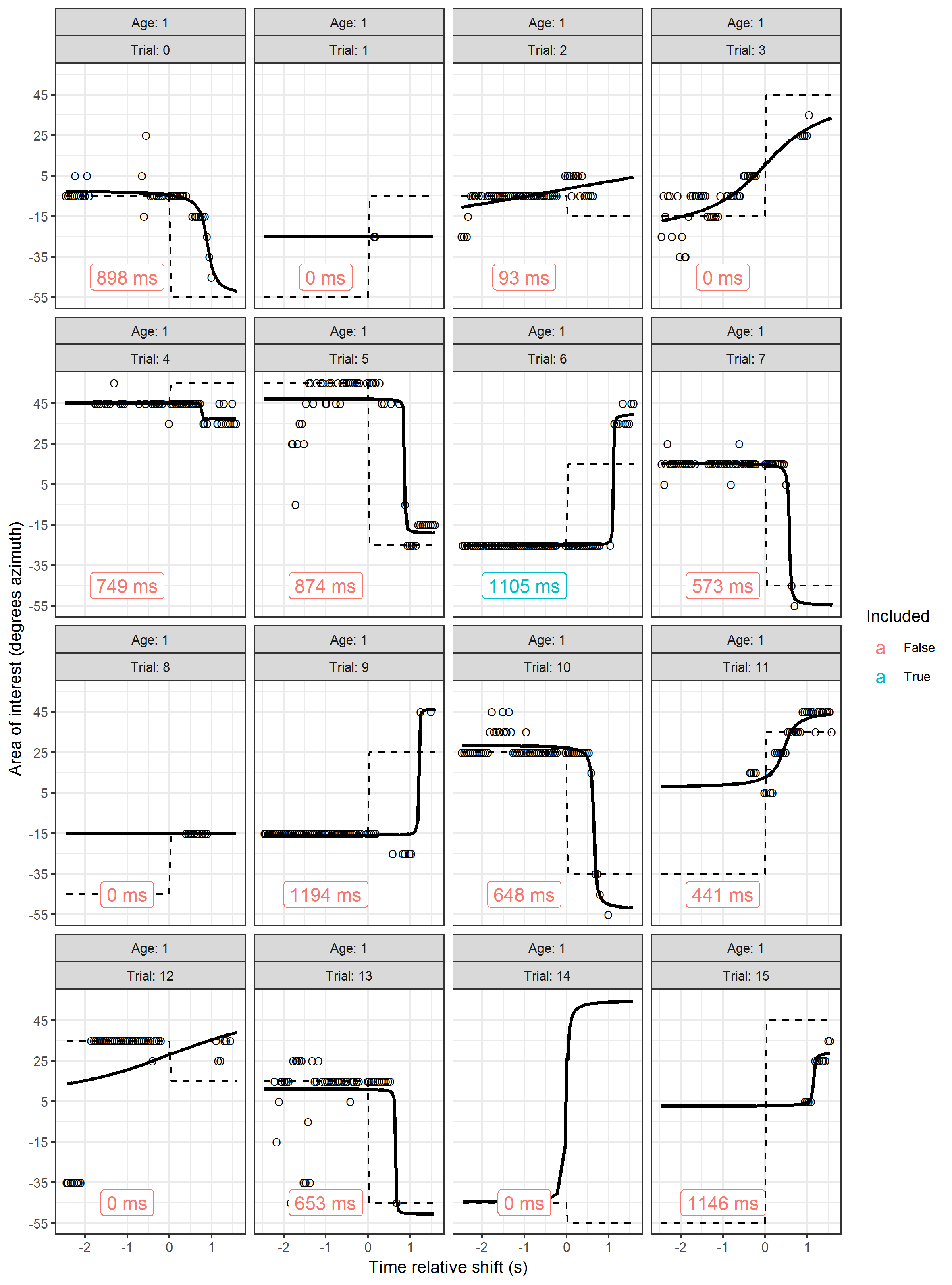

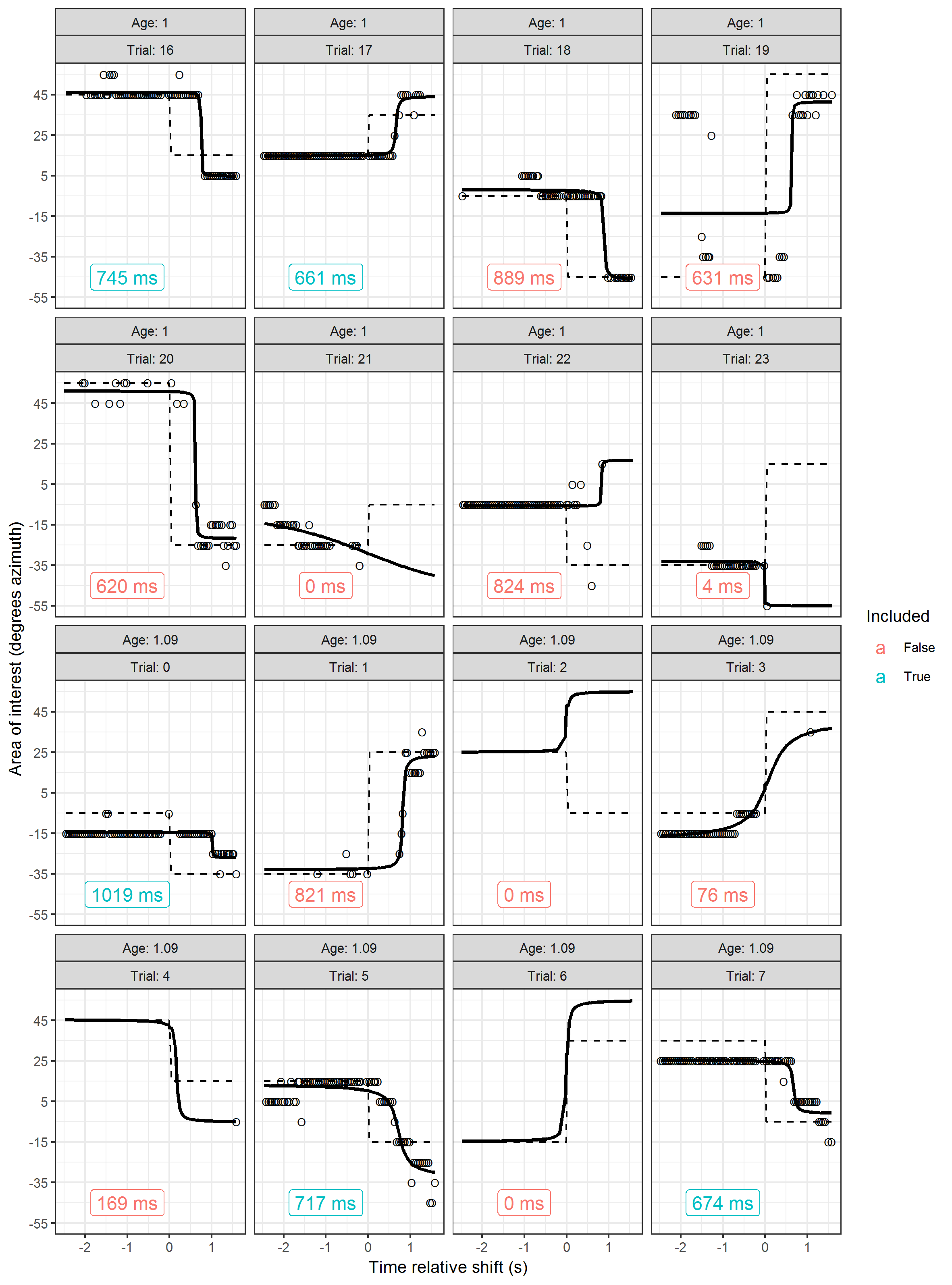

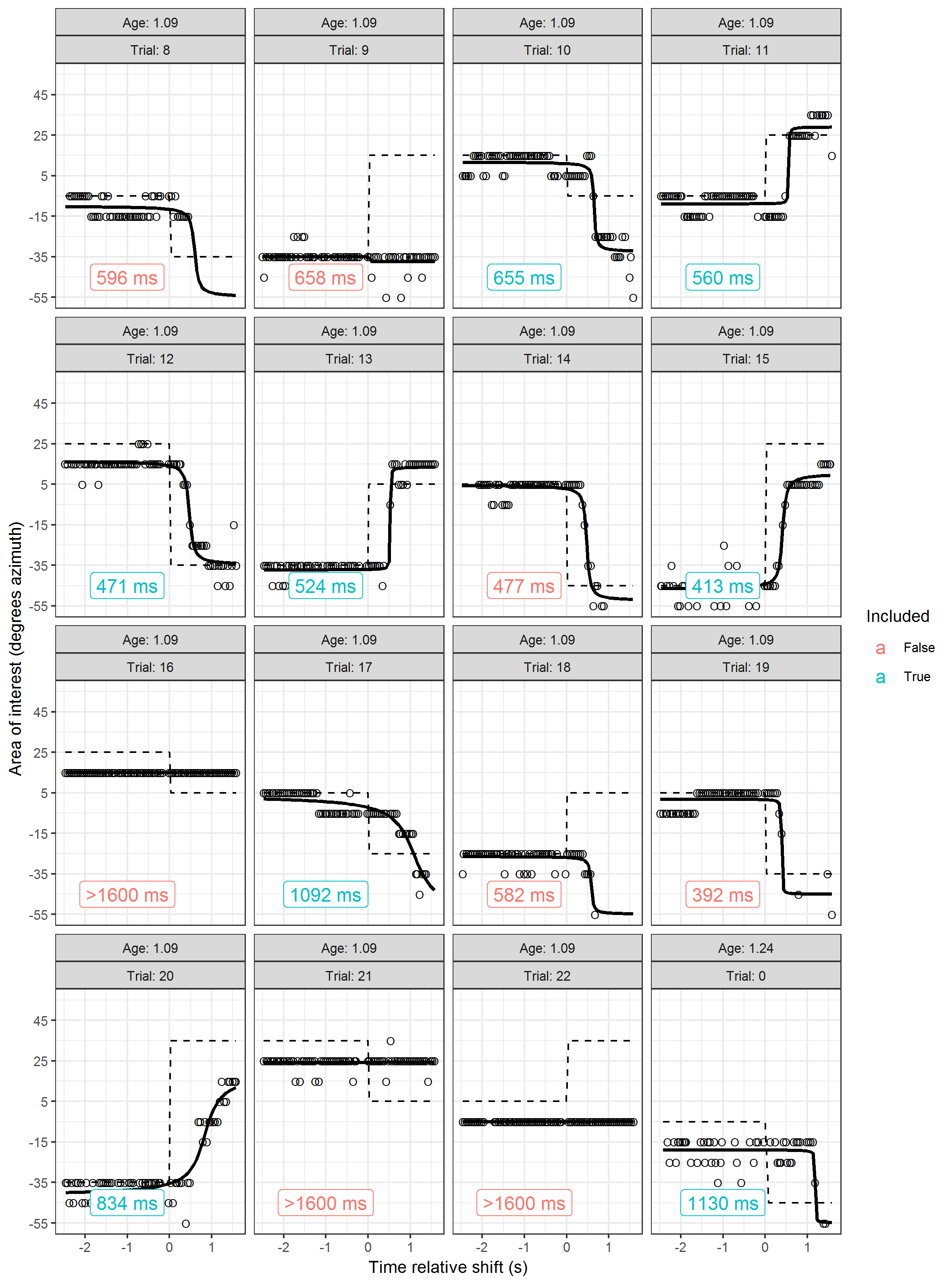

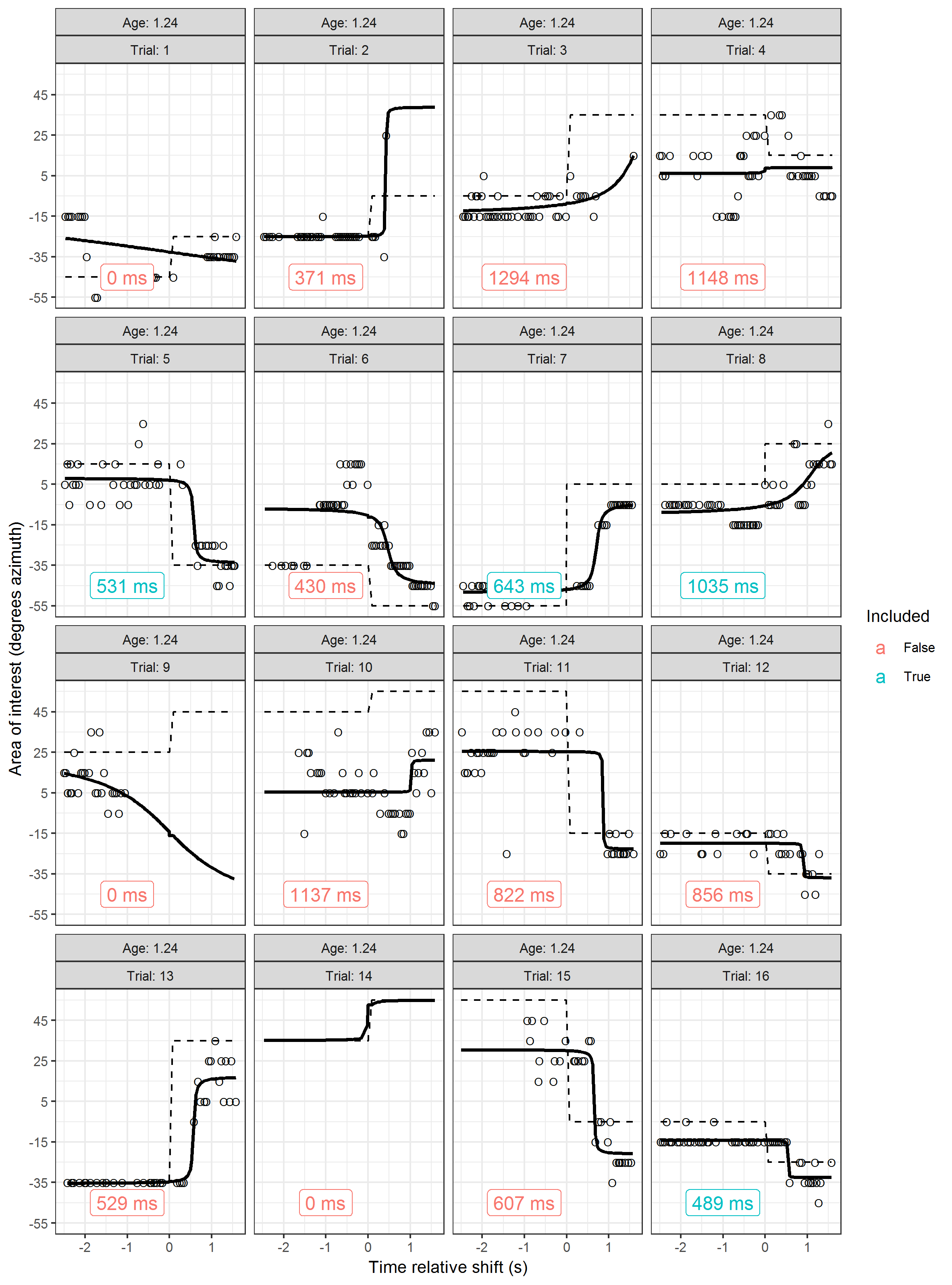

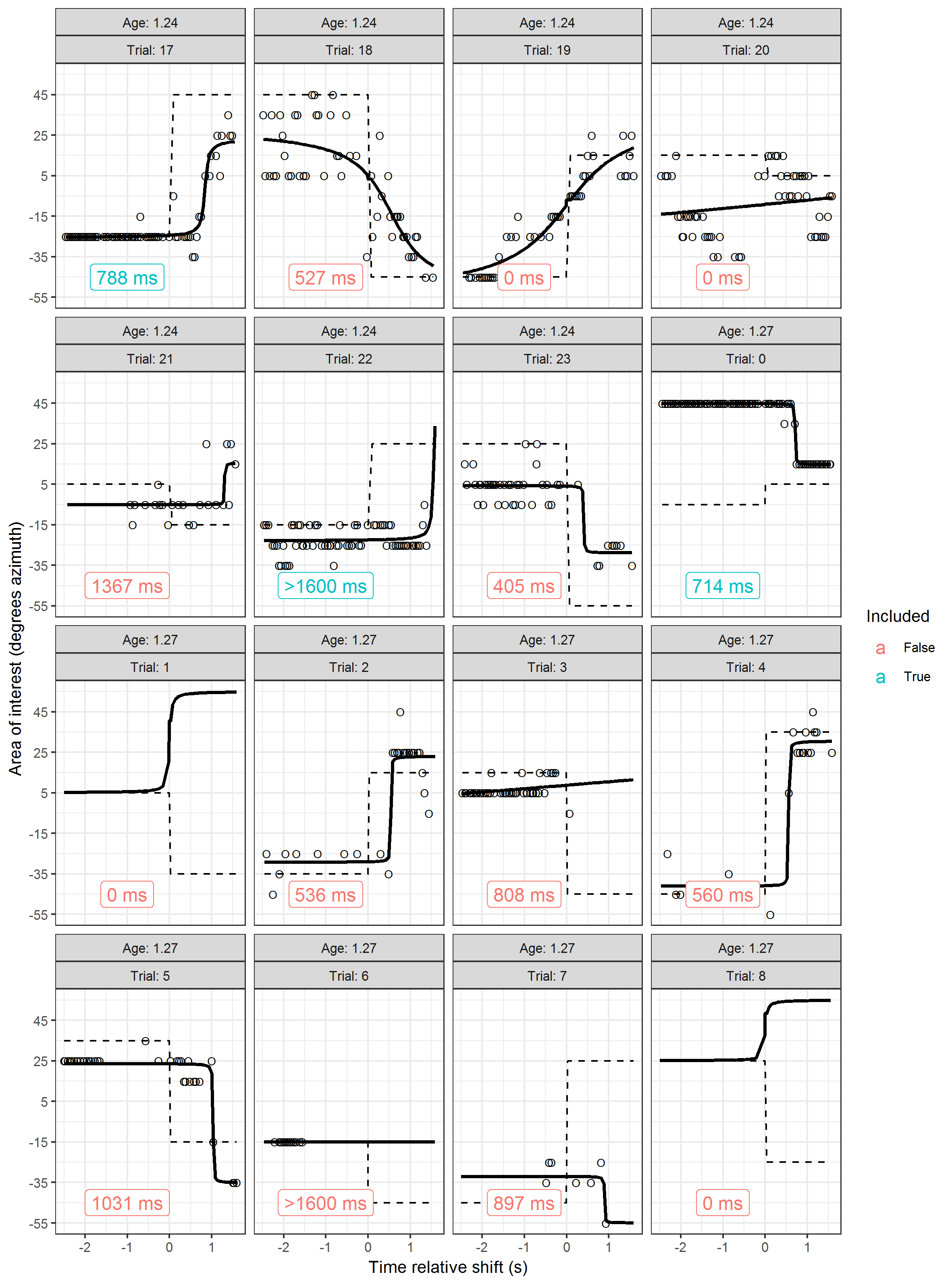

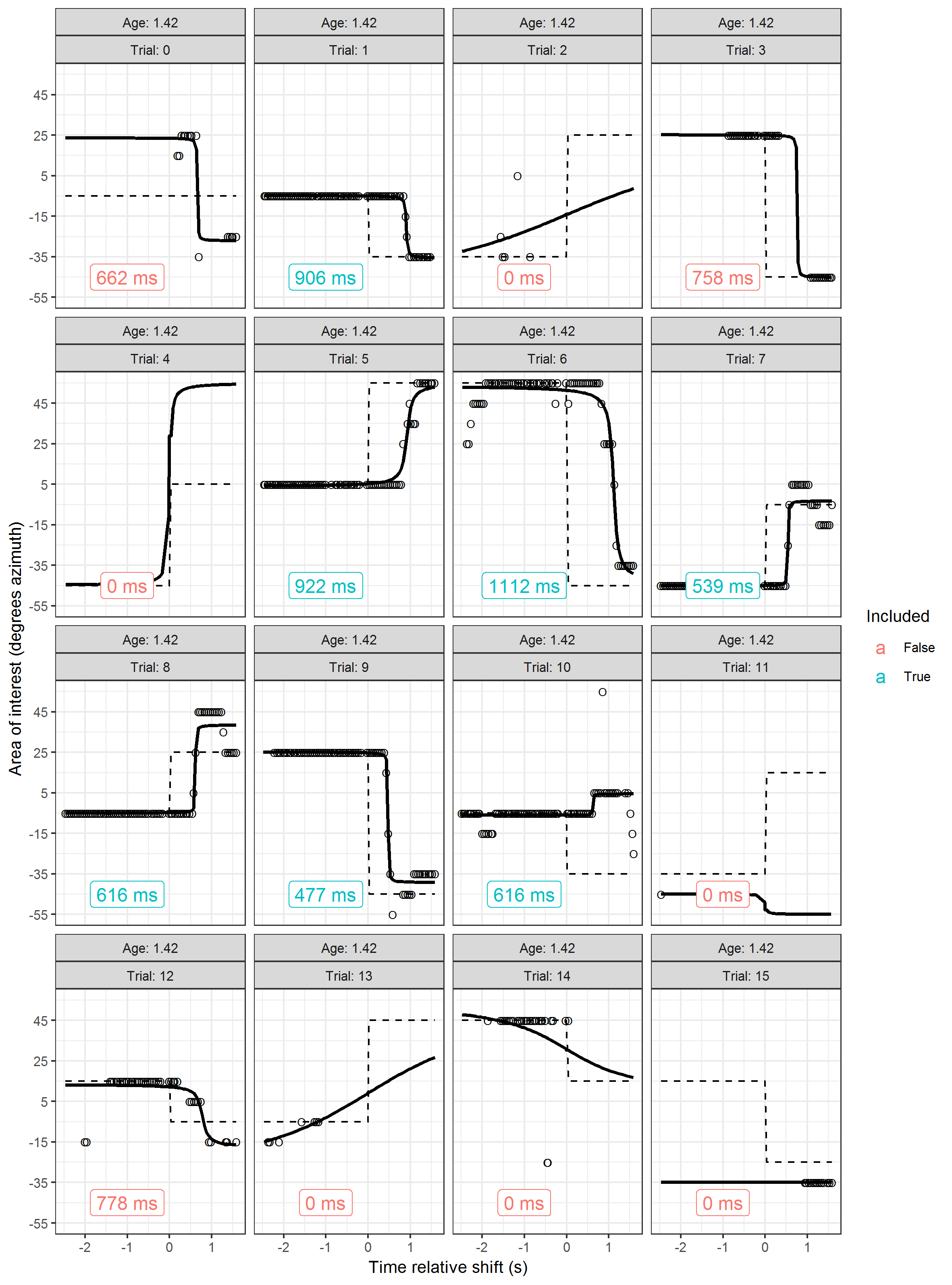

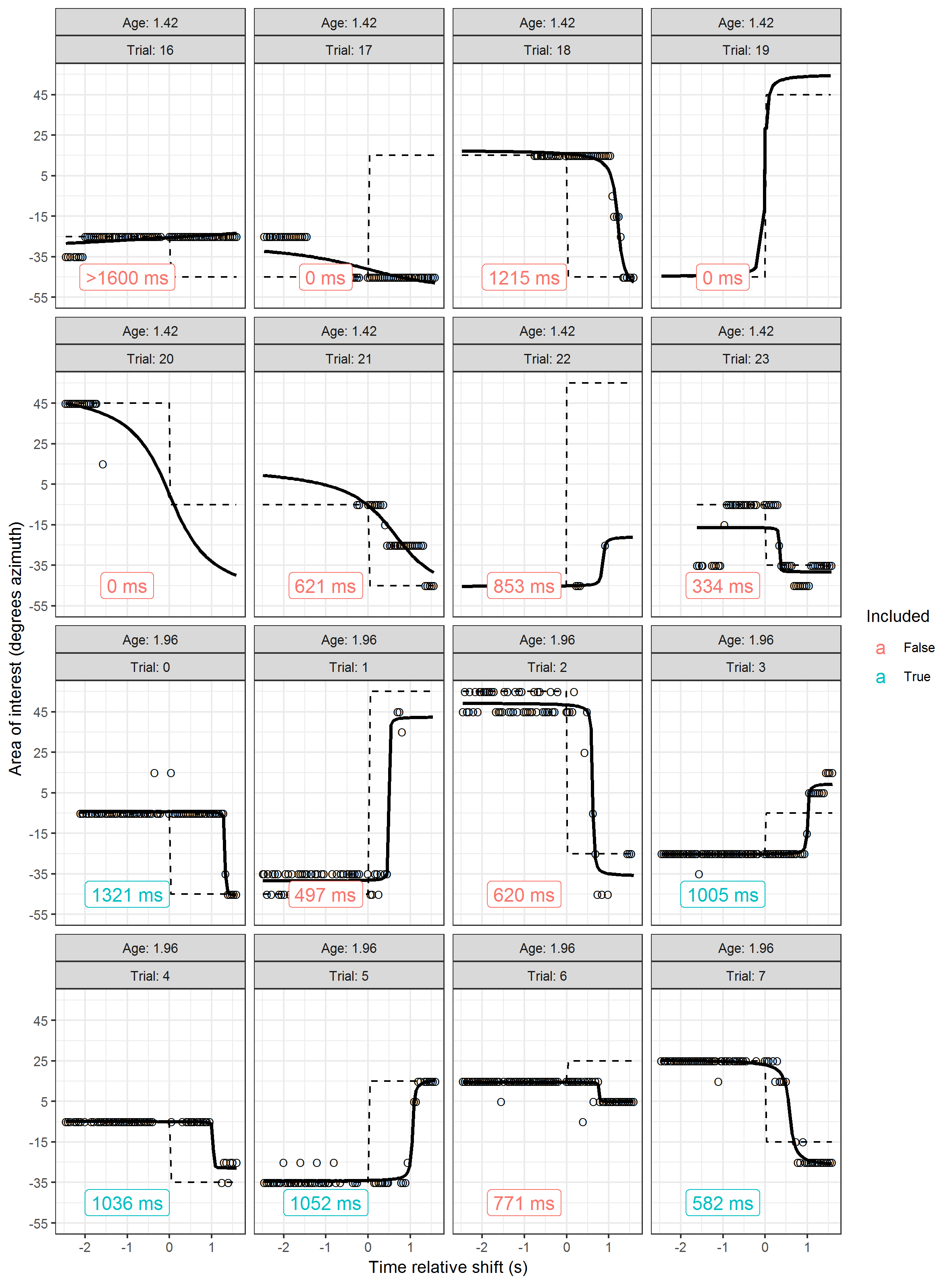

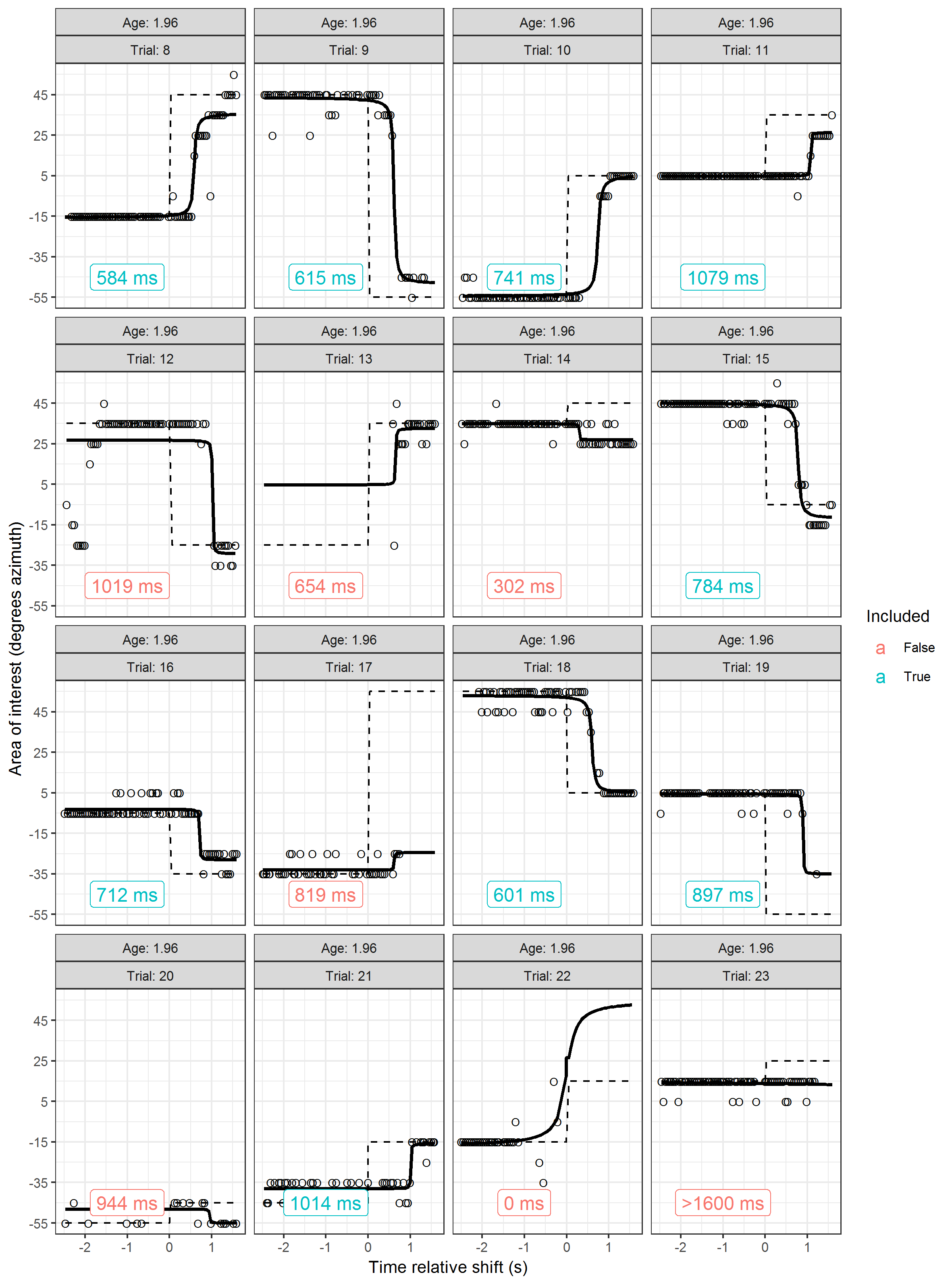

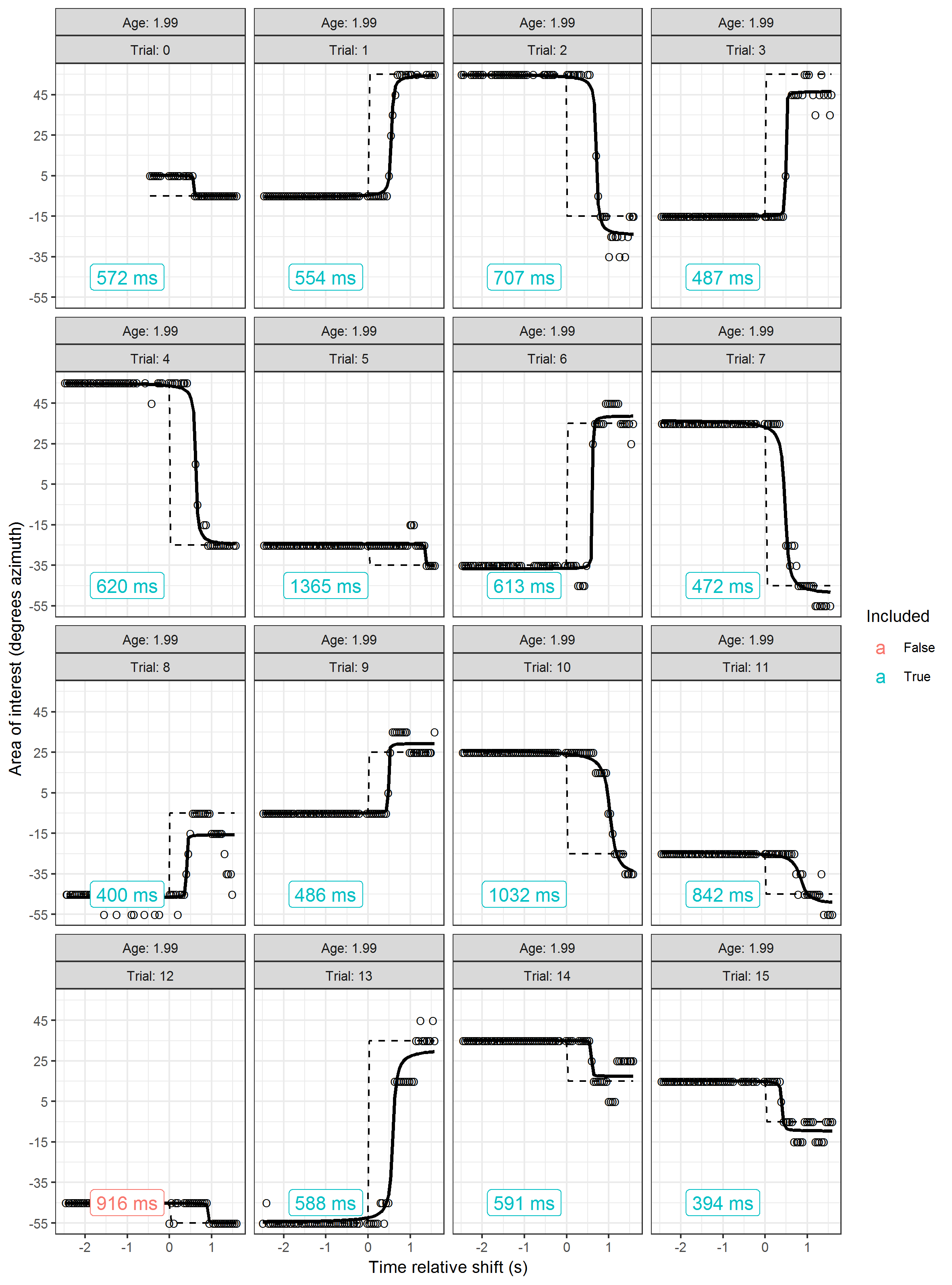

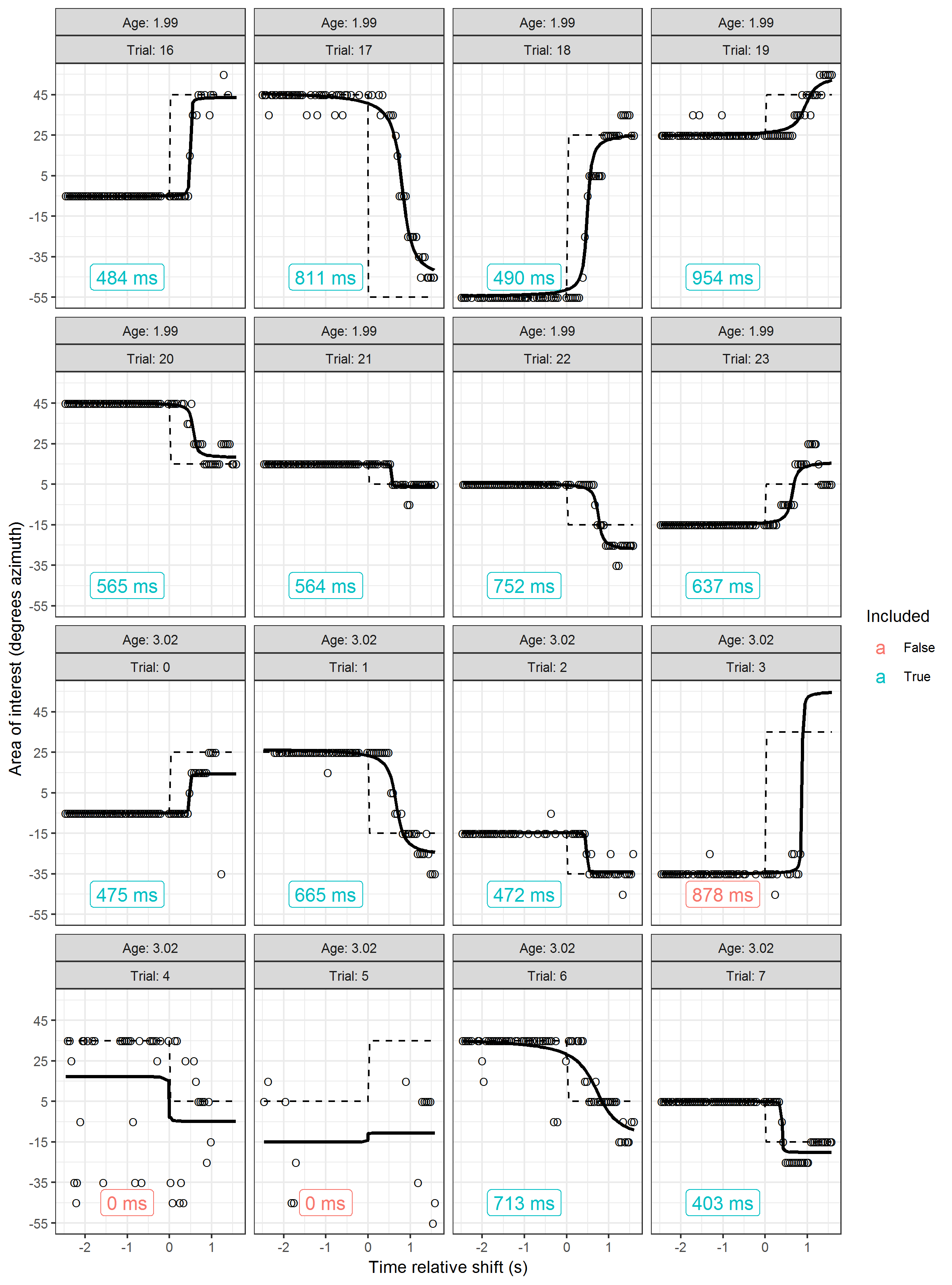

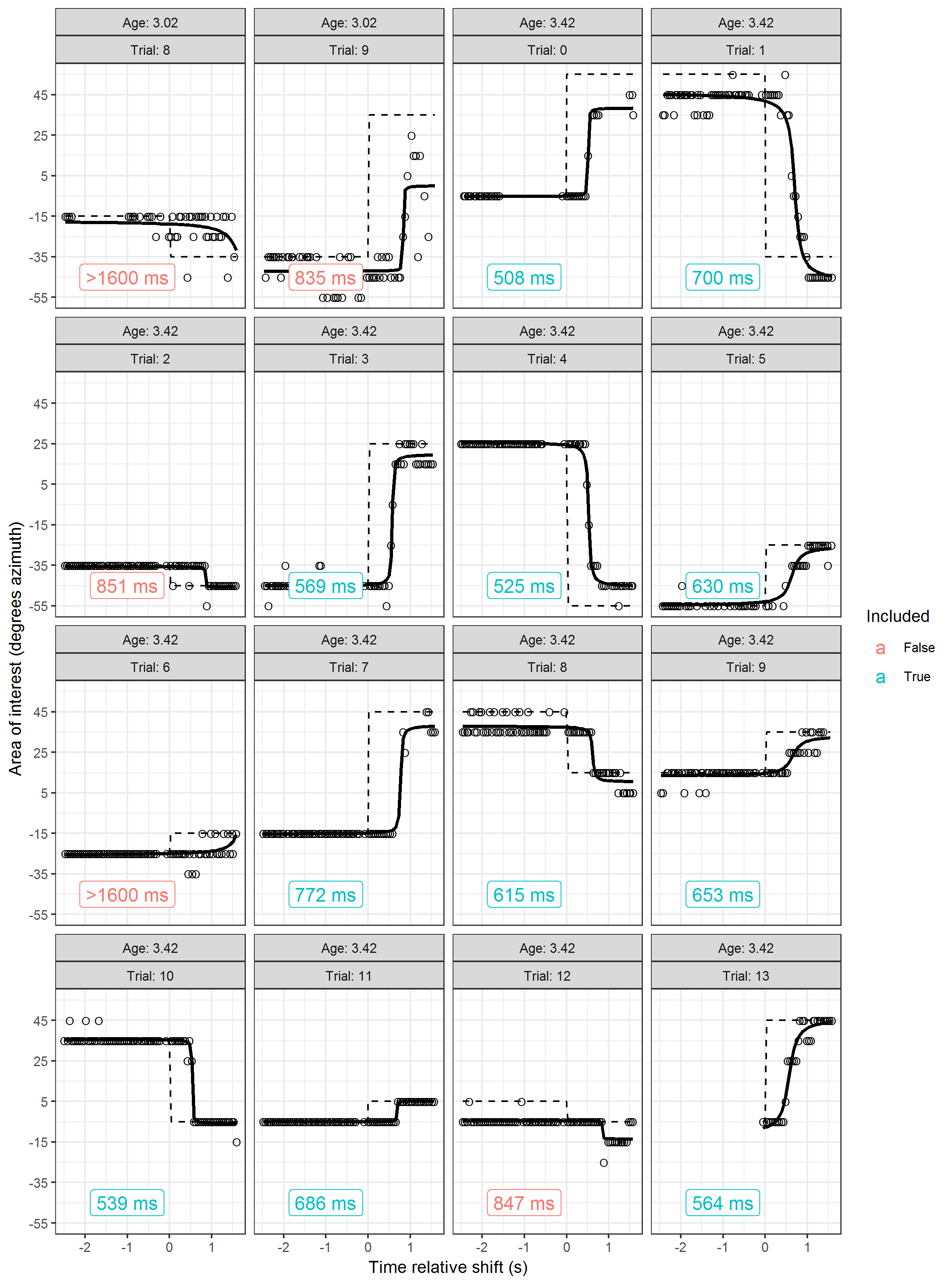

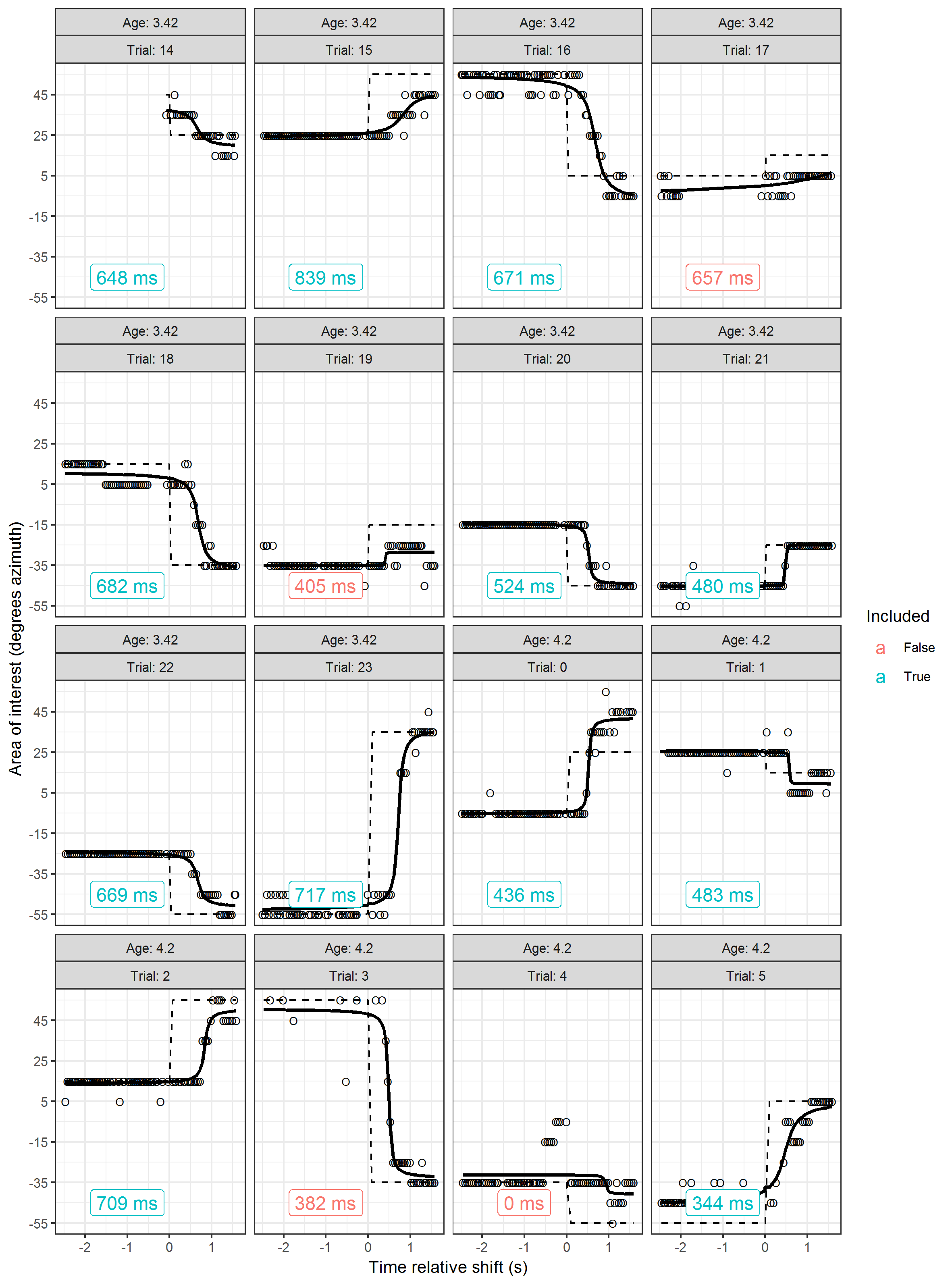

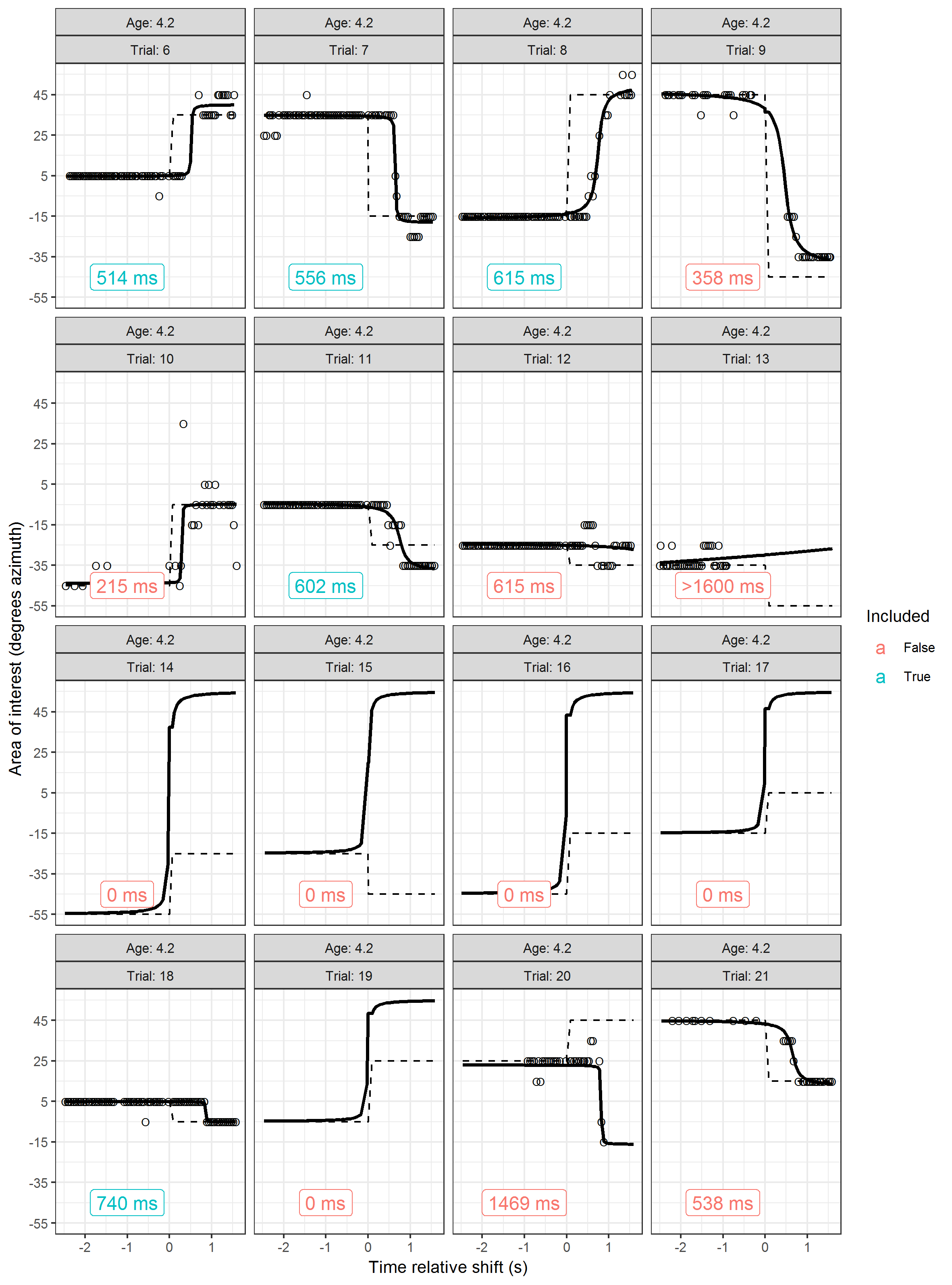

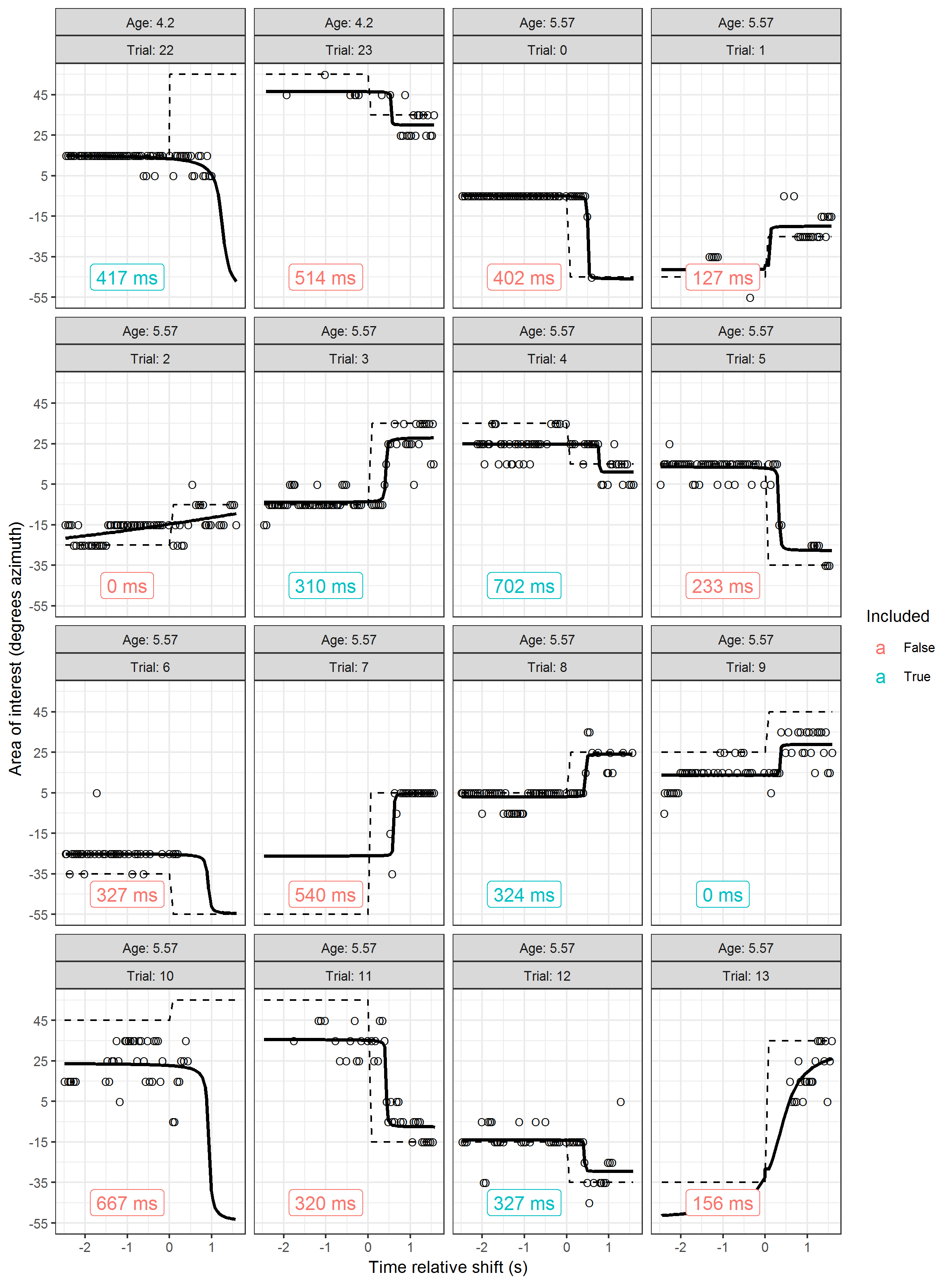

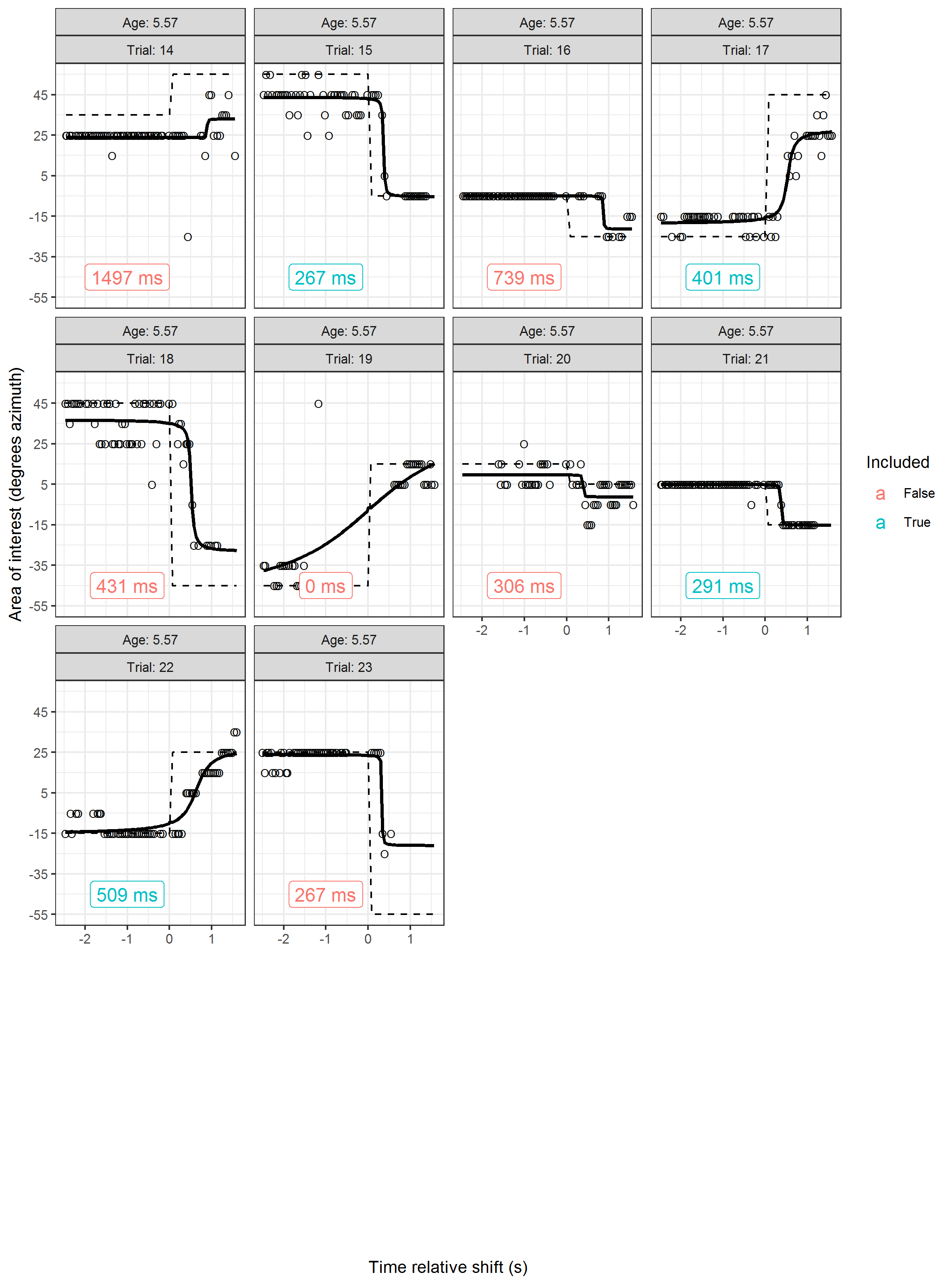


Figure S3. All trials from all subjects. Each subject is denoted by their age in decimal years at test and the participating trial below. The results from the objective exclusion criteria are shown by color.
